# Heterozygous variants in GATA2 contribute to DCML deficiency in mice by disrupting tandem protein binding

**DOI:** 10.1038/s42003-022-03316-w

**Published:** 2022-04-19

**Authors:** Atsushi Hasegawa, Yuki Hayasaka, Masanobu Morita, Yuta Takenaka, Yuna Hosaka, Ikuo Hirano, Masayuki Yamamoto, Ritsuko Shimizu

**Affiliations:** 1 Department of Molecular Hematology, Tohoku University Graduate School of Medicine, Sendai, 980-8575 Japan; 2 Tohoku Medical Mega-Bank Organization, Tohoku University, Sendai, 980-8575 Japan; 3 Department of Environmental Medicine and Molecular Toxicology, Tohoku University Graduate School of Medicine, Sendai, 980-8575 Japan; 4 Department of Medical Biochemistry, Tohoku University Graduate School of Medicine, Sendai, 980-8575 Japan

**Keywords:** Disease model, Leukopoiesis

## Abstract

Accumulating lines of clinical evidence support the emerging hypothesis that loss-of-function mutations of GATA2 cause inherited hematopoietic diseases, including Emberger syndrome; dendritic cell, monocyte B and NK lymphoid (DCML) deficiency; and MonoMAC syndrome. Here, we show that mice heterozygous for an arginine-to-tryptophan substitution mutation in GATA2 (*G2*^R398W/+^), which was found in a patient with DCML deficiency, substantially phenocopy human DCML deficiency. Mice heterozygous for the GATA2-null mutation (*G2*^-/+^) do not show such phenotypes. The G2^R398W^ protein possesses a decreased DNA-binding affinity but obstructs the function of coexpressed wild-type GATA2 through specific *cis*-regulatory regions, which contain two GATA motifs in direct-repeat arrangements. In contrast, G2^R398W^ is innocuous in mice containing single GATA motifs. We conclude that the dominant-negative effect of mutant GATA2 on wild-type GATA2 through specific enhancer/silencer of GATA2 target genes perturbs the GATA2 transcriptional network, leading to the development of the DCML-like phenotype. The present mouse model provides an avenue for the understanding of molecular mechanisms underlying the pathogenesis of GATA2-related hematopoietic diseases.

## Abstract (web summary)

DCML deficiency is a disorder marked by loss of multiple immune cell types. Mutations that affect a single allele of the *GATA2* transcription factor may lead to DCML by interfering with normal GATA2 binding, altering expression of important immune cell pathways.

## Introduction

GATA2 is a member of the GATA family of transcription factors that recognize the GATA consensus motif ((T/A)GATA(A/G)) and transcriptionally regulate the expression of target genes^1–3^. In mammals, 6 GATA factors have been identified, each of which has two highly conserved C2C2-type zinc finger domains. Of the zinc-finger domains, the C-terminal zinc finger (CF) domain is indispensable for binding to the GATA motif and is thus required for the transcriptional activity of GATA factors^4^. Among the six mammalian GATA factors, GATA1, GATA2, and GATA3 are hematopoietic GATA factors that were originally identified in hematopoietic cells. However, these factors are also expressed in organs/tissues other than those of the hematopoietic system. For instance, GATA2 has been found to contribute to the development of the urinary tract, endothelial cells and the nervous system^5–10^. Two *Gata2*-knockout alleles were generated: one was generated by replacing the 5th exon that encodes the CF domain with a neomycin-resistance cassette^11^, and the other was generated by inserting *Gfp* cDNA into the translation initiation site of the 2nd exon^12^. In both cases, homozygotes for the targeted allele died around embryonic day 10 (E10), the stage before the start of fetal liver hematopoiesis^11,12^. In addition, a floxed allele of GATA2 for conditional gene knockout has been generated and used for functional analyses of GATA2 during pituitary development^13^.

GATA2 plays important roles in the maintenance of hematopoietic stem cells (HSCs). In fact, severe HSC failure and depletion are induced by conditional deletion of the GATA2 CF domain in fetal and adult mice^14,15^. The expression of the *Gata2* gene changes dynamically during lineage differentiation hierarchy; therefore, GATA2 contributes to hematopoietic homeostasis, especially in the differentiation of granulocytes, mast cells, and dendritic cells^16–19^. An intriguing observation in this regard is that in the erythroid lineage, a complex regulatory network induces GATA2 expression in early progenitors to switch to GATA1 expression during differentiation, a process referred to as GATA factor switching^20–22^.

In a clinical study, a gain-of-function mutation caused by a single amino acid substitution in the CF domain of GATA2 was identified in myeloid transformation of chronic myelogenous leukemia^23^. This mutant GATA2 possesses increased transactivation activity along with enhanced inhibitory effects on the transcription factor PU.1. Soon after this discovery, many loss-of-function GATA2 mutations were identified that cause autosomal inherited and sporadic hematopoietic diseases^24–27^. Of note, heterozygotes harboring the latter *GATA2* gene mutations were found to exhibit features of various types of hematovascular-associated immunodeficiencies, such as Emberger syndrome, which displays primary lymphedema^25^; dendritic cell, monocyte B, and NK lymphoid (DCML) deficiency, which is characterized by decreases in circulating B cells (BCs), natural killer cells (NKCs), dendritic cells (DCs), and monocytes^26^; and MonoMAC syndrome, which is defined as DCML deficiency accompanying *Mycobacterium avium* complex infection^27^. Patients with these diseases have a high risk of developing myelodysplastic syndrome (MDS), acute myeloid leukemia (AML) and chronic myelomonocytic leukemia (CMML)^18,28^. Since the first discovery of inherited GATA2 mutation-related diseases, accumulating evidence has supported the notion that a variety of GATA2 mutations, not only missense and short indel mutations but also large deletions, splicing errors, gene-regulatory perturbations, frame shifts, and nonsense mutations, are involved in the pathogenesis of GATA2-related diseases^29^.

*GATA2* gene mutations can be divided into two types: one causes a reduction in *GATA2* expression (quantitative deficit), while the other leads to the production of structural GATA2 mutants (qualitative defect)^29^. In the latter type, mutations are found to accumulate in the CF domain and are predicted to cause a loss of DNA-binding activity and transactivation activity^29–31^. While characteristic differences in clinical outcomes have not yet been clarified between these two types of GATA2 mutations, higher disease penetrance and a higher incidence of leukemia transformation have been suggested in patients with qualitative defects than in patients with quantitative deficits^32,33^.

We aimed to investigate these GATA2-related diseases by establishing mouse models. In this regard, we maintained heterozygous mice carrying both types of *Gata2*-knockout alleles; they were apparently healthy and enjoyed a natural life. Neither type of mouse showed cytopenia, immunodeficiency, or lymphoedema. No published report has examined leukemogenesis in GATA2-heterozygous null mutant mice, but closer examinations revealed that the HSC function of these mice was slightly disrupted^34,35^. To further explore whether the quantitative deficit of GATA2 can serve as a model of human GATA2-related diseases, we established an alternative mouse model, a hypomorphic *G2*^fGN/fGN^ mutant mouse line, in which the expression level of GATA2 was reduced to as low as 20% of the endogenous GATA2 level^9,36^. While approximately 70% of *G2*^fGN/fGN^ mice died soon after birth due to urogenital deformity, mice that survived adolescence were prone to develop myeloproliferative neoplasms (MPNs) in the adult stage, with features resembling human CMML^36^. Thus, although quantitative GATA2 deficits convert immature myeloid progenitors to cells that easily expand and generate many descendants, these deficits do not induce cytopenia in BCs, NKCs, DCs or monocytes, which are hallmarks of GATA2 mutation-related diseases, indicating that quantitative deficits of GATA2 are not a good model of GATA2 mutation-related diseases.

Therefore, in this study, we decided to establish a mouse line harboring a qualitative defect of GATA2, hoping to recapitulate the clinical GATA2 mutant-related phenotypes. To this end, we generated mice that expressed the mutant GATA2 protein in which arginine 398 was substituted for tryptophan (G2^R398W^). We found that heterozygous expression of G2^R398W^ indeed leads to age-progressive multilineage cytopenia of BCs, NKCs, DCs and monocytes, which phenocopies human DCML deficiency, demonstrating that the G2^R398W^ heterozygote serves as a valuable model of human GATA2 mutation-related diseases. Importantly, we also clarified that G2^R398W^ and another mutant GATA2 protein, G2^T354M^, have a dominant-negative effect on the native GATA2 protein in terms of DNA-binding activity for the specific *cis*-element configuration. We propose that this dominant-negative effect induces perturbations of the regulation of specific GATA2 target genes, which explains, at least in part, the development of the DCML-deficient-like phenotype.

## Results

### Generation and characterization of *G2*^R398W^ mutant mice

**Fig. 1:**
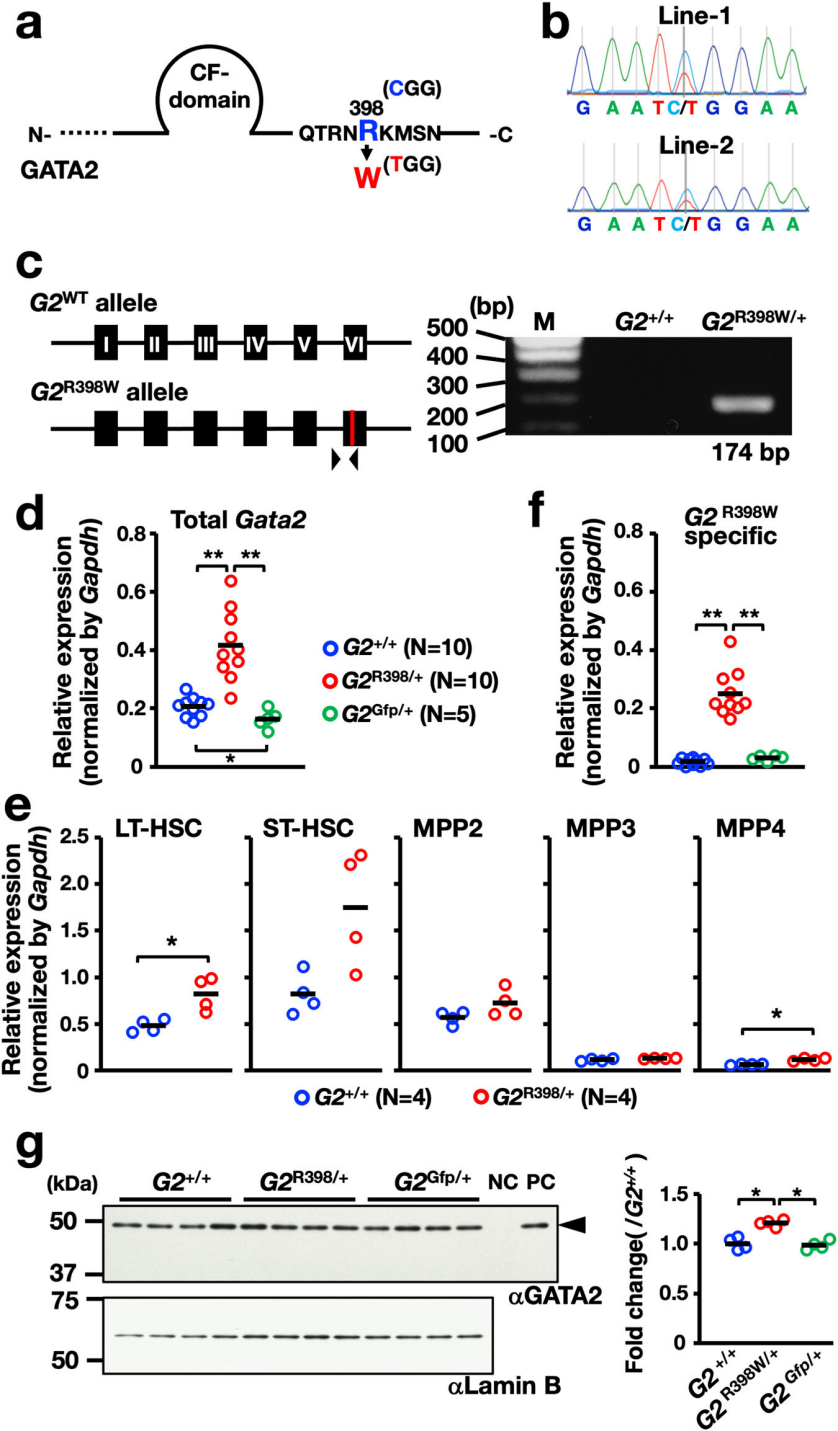
Generation of genome-edited mice carrying the *G2*^R398W^ mutation. **a** Schematic illustration of genome editing patterns and locations. **b** Genomic DNA sequences of two independent founder mice at the targeted region of the *Gata2* gene. The signal peaks of the original cytosine (blue) and substituting thymine (red) overlap at position c.1192. **c** PCR-based genotyping strategy of the *G2*^R398W^ allele containing the substituted nucleotide. c.1192C>T in the 6th exon is indicated as a red line. Positions recognized by the forward and reverse primers are indicated as arrowheads. **d**, **e** Dot plots and mean values for *Gata2* transcripts expressed in LSK cells (**d**) and in subpopulations of LSK cells (**e**). A primer set detecting transcripts originating from both intact and mutated alleles was used. **f** Dot plots and mean values for mutated *Gata2* transcripts expressed in LSK cells, which were detected using a mutated allele-specific primer set. Number of mice are indicated in parentheses. **p < 0.01. **g** Immunoblot analysis using nuclear extracts from bone marrow cells. Four independent mice of each genotype were used. Cell extracts from original and murine GATA2-expressing HEK293T cells were used as negative (NC) and positive (PC) controls, respectively. The levels of GATA2 were evaluated by comparing the signal intensity with that of Lamin B. The average value of GATA2 in *G2*^+/+^ mice was set to 1.0. The average and individual relative values of GATA2 in individuals are shown in the right panel. *p < 0.05.

We established GATA2 mutant mouse lines in which arginine 398 of GATA2 was substituted for tryptophan (R398W) by the CRISPR/Cas9 technique (Fig. 1a). This type of mutation corresponds to the human G2^R398W^ mutation that is frequently found in inherited GATA2-related diseases, such as DCML deficiency, MonoMAC syndrome and Emberger syndrome^29^. We established two founder mice carrying a single-nucleotide c.1192C>T substitution mutation at the *Gata2* locus (Fig. 1b) and crossed these founder mice with wild-type mice to generate heterozygous mutant mice (*G*2^R398W/+^). The mice were genotyped by using PCR with genotype-specific PCR primer sets (Fig. 1c and Supplementary Fig. 1a). As the two founder mice did not show any apparent difference, we utilized mainly Line-1 offspring for further analyses. Heterozygous *G2*^R398W/+^ pups generated by crossing *G2*^R398W/+^ and *G2*^+/+^ mice were born according to the Mendelian ratio (73 out of 156 newborns) and did not display any growth differences from their wild-type littermates.

We performed quantitative RT-PCR (qRT-PCR) analysis to determine the total transcript expression level of *Gata2* (wild-type plus mutant) in bone marrow Lineage^–^Sca1^+^cKit^+^ (LSK) cells from *G2*^R398W/+^ mice by utilizing primers covering exons 5 and 6. To our surprise, the total transcript expression level of *Gata2* in the *G2*^R398W/+^ mice was significantly increased approximately 2.0-fold on average compared with that in the *G2*^+/+^ mice, while the expression level of *Gata2* in heterozygous *Gata2* knockout mice (*G2*^Gfp/+^)^12^ carrying the heterozygous *Gfp* knockin-knockout allele in the *Gata2* locus (*G2*^Gfp^ allele) was slightly but significantly decreased by ~0.8-fold on average compared with that in the *G2*^+/+^ mice (Fig. 1d). We subdivided LSK cells into long-term (LT)-HSCs, short-term (ST)-HSCs, multipotent progenitor 2s (MPP2s), MPP3s and MPP4s and found that the increase in *Gata2* expression was striking in LT-HSCs of *G2*^R398W/+^ mice, in which the *Gata2* expression was high (Fig. 1e). We confirmed that the expression of transcripts derived from the mutated allele was specifically identified in *G2*^R398W/+^ mice (Fig. 1f), which indicated that mRNA transcription of *Gata2* from the mutant *Gata2* locus is active.

We then examined the total protein expression levels of GATA2 in the bone marrow of mice by immunoblotting analysis. Consistent with the qPCR analyses, the GATA2 protein level in the *G2*^R398W/+^ mice was elevated approximately 1.2-fold compared to that in *G2*^+/+^ mice, while changes in the level of GATA2 protein in *G2*^Gfp/+^ mice were not remarkable (Fig. 1g and Supplementary Fig. 1b). These results demonstrate that GATA2 expression is significantly increased in the bone marrow of *G2*^R398W/+^ mutant mice. However, the mechanisms underlying this upregulation of *Gata2* gene expression remain elusive.

### Activity of the G2^R398W^ mutation during mouse embryogenesis

**Fig. 2:**
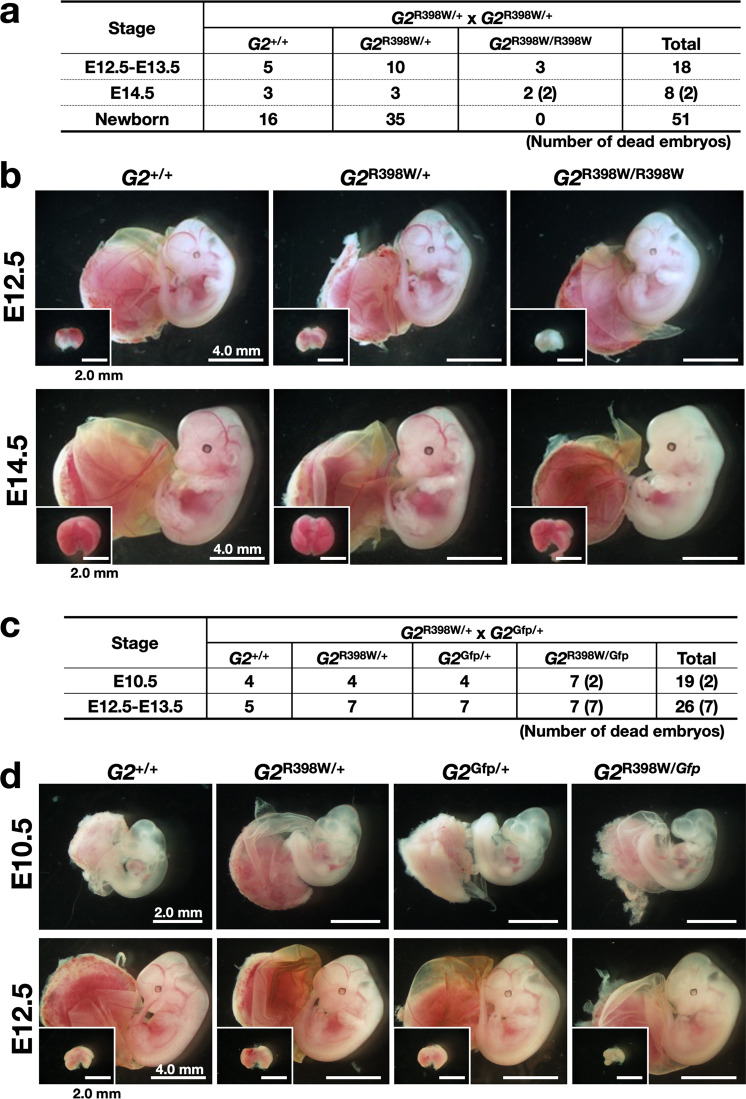
Allele number-dependent effect of *G2*^R398W^ on mouse embryogenesis. Intercrossing of *G2*^R398W/+^ mice (**a**, **b**) and mating of fetuses with *G2*^Gfp/+^ mice (**c**, **d**). The numbers of embryos of each genotype at the indicated stage are shown (**a**, **c**), along with representative photos (**b**, **d**). Livers are shown in the insets (**b**, **d**). The number of dead embryos is indicated by parentheses. Note that no live *G2*^R398W/Gfp^ embryo was found at E12.5-E13.5.

To explore the extent to which the G2^R398W^ mutant protein lost its activity and whether the mutant protein could support mouse embryogenesis, we intercrossed *G2*^R398W/+^ mice. We found that homozygous *G2*^R398W/R398W^ embryos were born following the Mendelian ratio and survived beyond E12.5. However, the homozygous mice died around E14.5, and no homozygous newborns were obtained (Fig. 2a, b). As the critical developmental stage in *Gata2*-knockout mice is between E10.5 and E11.5^11^, these results demonstrate that the G2^R398W^ mutant protein retains GATA2 activity to a certain extent and is able to support mouse embryogenesis until E14.5.

To further examine G2^R398W^ mutant protein activity in the context of mouse development, we next crossed the *G2*^R398W/+^ mice with the *G2*^Gfp/+^ mice and found that compound heterozygous embryos carrying the *G2*^R398W^ and *G2*^Gfp^ alleles (*G2*^*R*398W/Gfp^) died by E12.5 (Fig. 2c, d), while heterozygotes of the *G2*^R398W^ and *G2*^Gfp^ alleles survived these stages and were born normally. These results indicate that the expression of the G2^R398W^ mutant protein contributes to embryonic development. The difference in the survival of *G2*^R398W/R398W^ embryos and *G2*^R398W/Gfp^ embryos further supports the hypothesis that the contribution of this protein occurs in an allele number-dependent manner.

### Appearance of DCML-deficient phenotypes in aged *G2*^R398W/+^ mice

We aimed to examine whether heterozygous *G2*^R398W/+^ mice showed phenotypes that recapitulate those of human diseases heterozygous for the GATA2 mutant protein, which include Emberger syndrome, DCML deficiency, and MonoMAC syndrome^29^. Therefore, to explore the disease-related phenotypes in *G2*^R398W/+^ mice, we analyzed hematopoietic features in aged adult mice. To this end, we first examined the hematological phenotypes of *G2*^R398W/+^ mice at 12 months compared to those of *G2*^+/+^ siblings.

**Fig. 3:**
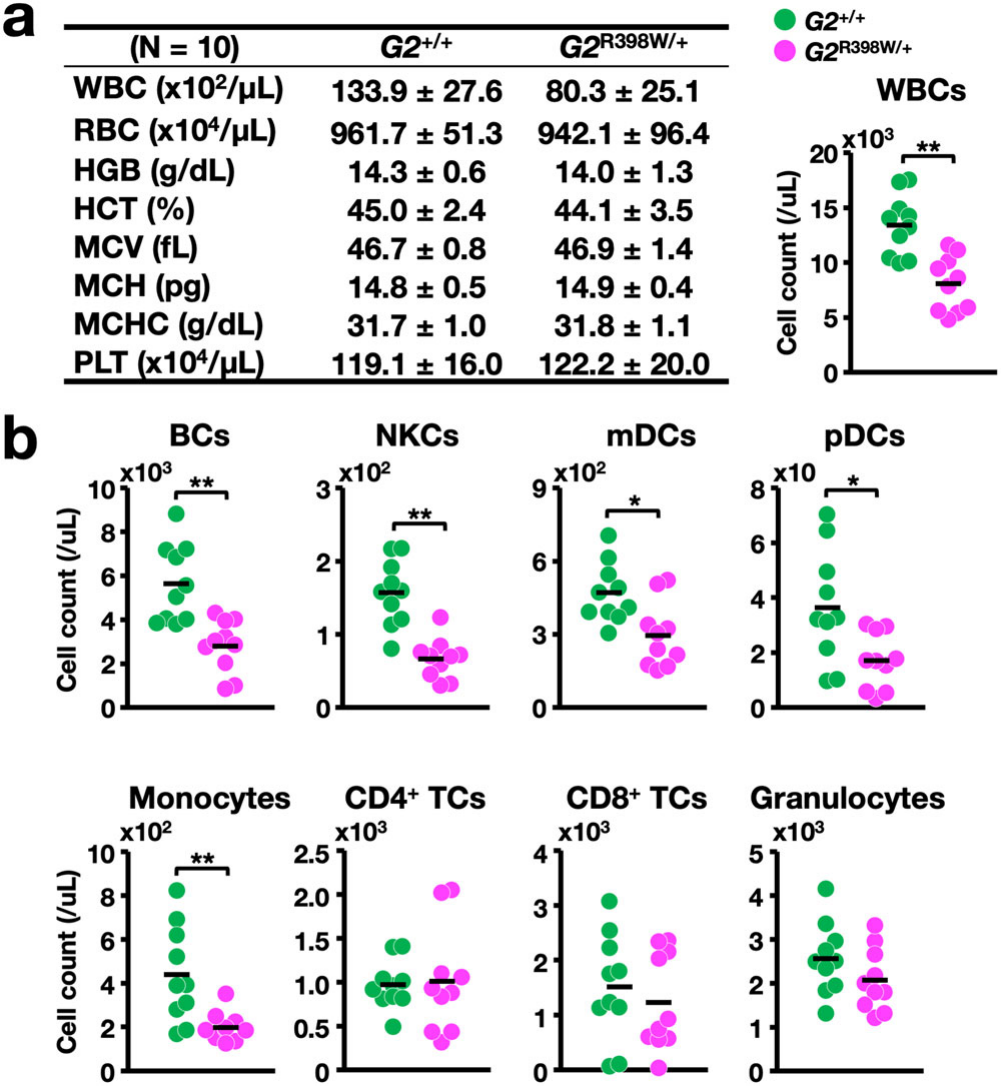
Evaluation of the peripheral blood counts of 12-month-old *G2*^R398W/+^ mice. **a** Summary of hematopoietic indices. A dot plot of WBC counts in the peripheral blood of *G2*^*+*/+^ and *G2*^R398W/+^ mice is shown in the right panel. **b** Dot plots of the cell counts of the indicated cell populations of *G2*^*+*/+^ and *G2*^R398W/+^ mice. *p < 0.05, **p < 0.01. *N* = 10 of each genotype.

In the evaluation of peripheral blood counts, we found that the white blood cell (WBC) count was significantly decreased in *G2*^R398W/+^ mice at 12 months compared with that in *G2*^+/+^ mice, while the red blood cell (RBC) and platelet counts were maintained in the normal range (Fig. 3a). Notably, BCs, NKCs, myeloid DCs (mDCs), and plasmacytoid DCs (pDCs) were significantly reduced in number in *G2*^R398W/+^ mice (Fig. 3b). Monocytopenia was also observed in the *G2*^R398W/*+*^ mice, but the numbers of CD4 single-positive T cells (CD4^+^ TCs), CD8 single-positive T cells (CD8^+^ TCs) and granulocytes in mutant mice were comparable to those in control mice and were maintained within the normal range (Fig. 3b). These phenotypes of the *G2*^R398W/+^ mice nicely recapitulate the clinical observations of the diseases caused by heterozygous GATA2 mutations, including R398W^29^, and indicate that the *G2*^R398W/+^ mice serve as an excellent disease model mouse line.

To verify this conclusion, we next analyzed younger generations of *G2*^R398W/+^ mice. To this end, we exploited 5- to 7-month-old and 3-month-old *G2*^R398W/+^ mice and examined hematopoietic indices of the peripheral blood. We found similar decreases in the numbers of WBCs, BCs, NKCs, mDCs, and pDCs in 5- to 7-month-old *G2*^R398W/+^ mice, but monocytopenia was not observed in the mice (Supplementary Fig. 2). Decreases in WBCs, BCs, NKCs, mDCs, pDCs, and monocytes were not observed in 3-month-old *G2*^R398W/+^ mice (Supplementary Fig. 3). The numbers of CD4^+^ TCs, CD8^+^ TCs, and granulocytes in the *G2*^R398W/+^ mice were comparable to those in control mice at both the 5-7-month and 3-month time points. We further examined the second line of *G2*^R398W/+^ mice and found similar decreases in WBC count and WBC subpopulations in the *G2*^R398W*/+*^ mouse line at 6 months of age (Supplementary Fig. 4).

### Partial decrease in hematopoietic stem and progenitor populations in *G2*^R398W/+^ mice

**Fig. 4:**
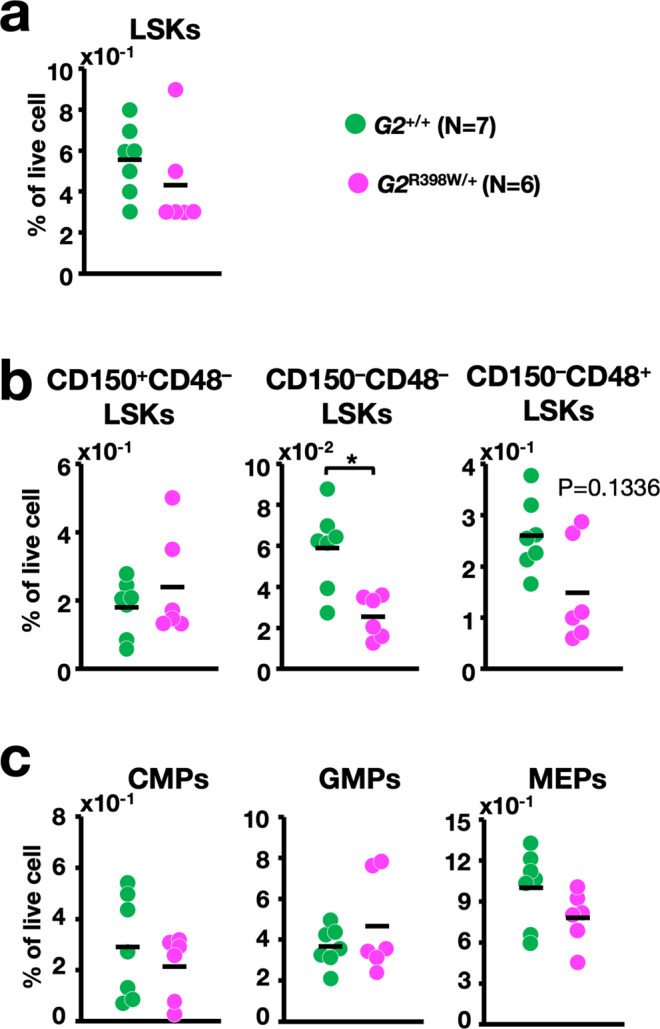
Born marrow stem and progenitor cell populations in 12-month-old *G2*^R398W/+^ mice. **a** Quantification of LSKs in the bone marrow of *G2*^*+*/+^ and *G2*^R398W/+^ mice. **b**, **c** Quantification of stem cell populations subdivided according to CD150 and CD48 profiles (**b**) and progenitor populations (**c**) in the bone marrow of *G2*^*+*/+^ and *G2*^R398W/+^ mice. *p < 0.05. *N* = 10 of each genotype.

As loss-of-function germline GATA2 mutations have been found in myelodysplastic syndrome and leukemias in humans^29^, we also examined bone marrow progenitors in *G2*^R398W/+^ mice. We analyzed the hematopoietic stem and progenitor cells of 12-month-old mice and found that the frequency of LSK in the *G2*^R398W/+^ mice was comparable to that in the *G2*^+/+^ siblings (Fig. 4a). In contrast, when we examined the LSK subpopulations, we found that the frequency of CD150^-^CD48^-^LSKs was significantly decreased (Fig. 4b).

We also examined the frequencies of myeloid-restricted progenitors, e.g., common myeloid progenitors (CMPs), granulocyte-macrophage progenitors (GMPs), and megakaryocyte-erythroid progenitors (MEPs). The frequencies of these progenitors did not change between *G2*^*R398W/+*^ and *G2*^+/+^ mice (Fig. 4c). During the course of the 12-month follow-up period, we carefully checked for the onset of leukemia, but we could not detect the development of hematological neoplasms in the *G2*^R398W/+^ mice. Thus, these results imply that while heterozygous status for the *G2*^*R398W*^ allele affects the differentiation and/or maintenance of hematopoietic progenitors, this influence is not strong enough to drive the development of hematopoietic malignancies.

### The *G2*^R398W/+^ hematopoietic phenotype does not exist in *G2*^Gfp/+^ mice

We next examined whether the hematopoietic phenotypes observed in the *G2*^R398W/+^ mice could be seen in heterozygous GATA2-knockout mice. For this purpose, we used the *Gfp* knockin/*Gata2* knockout mouse line at the 6-7-month timepoint^12^. We found that the analyses of peripheral blood hematopoietic indices and cell populations in the heterozygous *Gata2*-knockout (*G2*^Gfp/+^) mice generated results that stood in stark contrast to those of the *G2*^R398W/+^ mice.

**Fig. 5:**
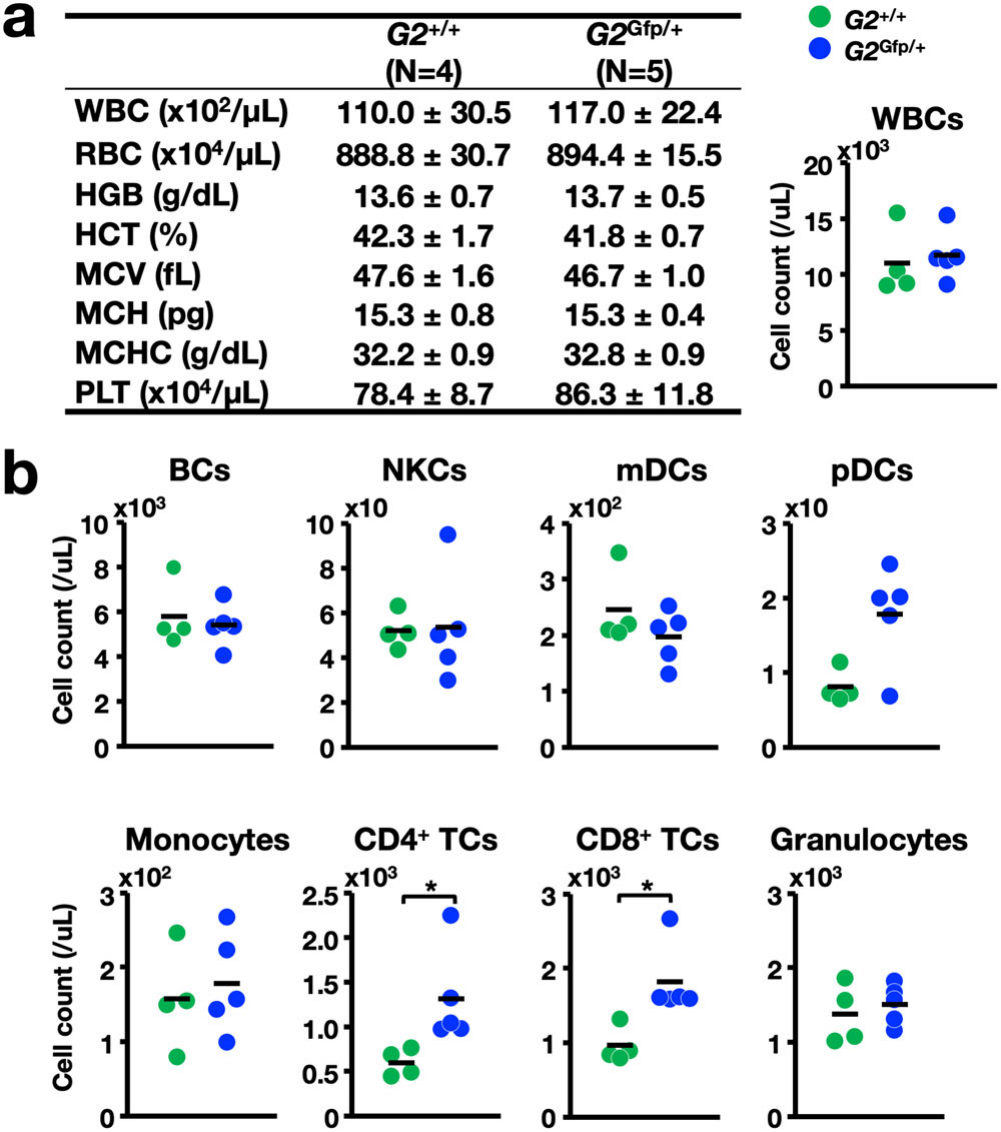
Evaluation of the peripheral blood counts of *G2*^Gfp/+^ mice at 6-7 months old. **a** Summary of hematopoietic indices. A dot plot of WBC counts in the peripheral blood of *G2*^*+*/+^ and *G2*^R398W/+^ mice and their *G2*^*+*/+^ littermates is shown in the right panel. **b** Dot plots of the cell count of the indicated cell population in the peripheral blood of *G2*^*+*/+^ and *G2*^R398W/+^ mice. *p < 0.05, *N* = 4–5 of each genotype.

The WBC numbers were comparable between the mice of these two genotypes (Fig. 5a), in good agreement with a previous report^34^. The numbers of BCs, NKCs, mDCs, pDCs, and monocytes did not change substantially (Fig. 5b). Therefore, these results support the hypothesis that the heterozygous G2^R398W^ mutation-induced hematological phenotypes, which are substantially different from those induced by the heterozygous GATA2-null mutation.

### GATA2 dimerizes regardless of mutations at residues R398 and T354

**Fig. 6:**
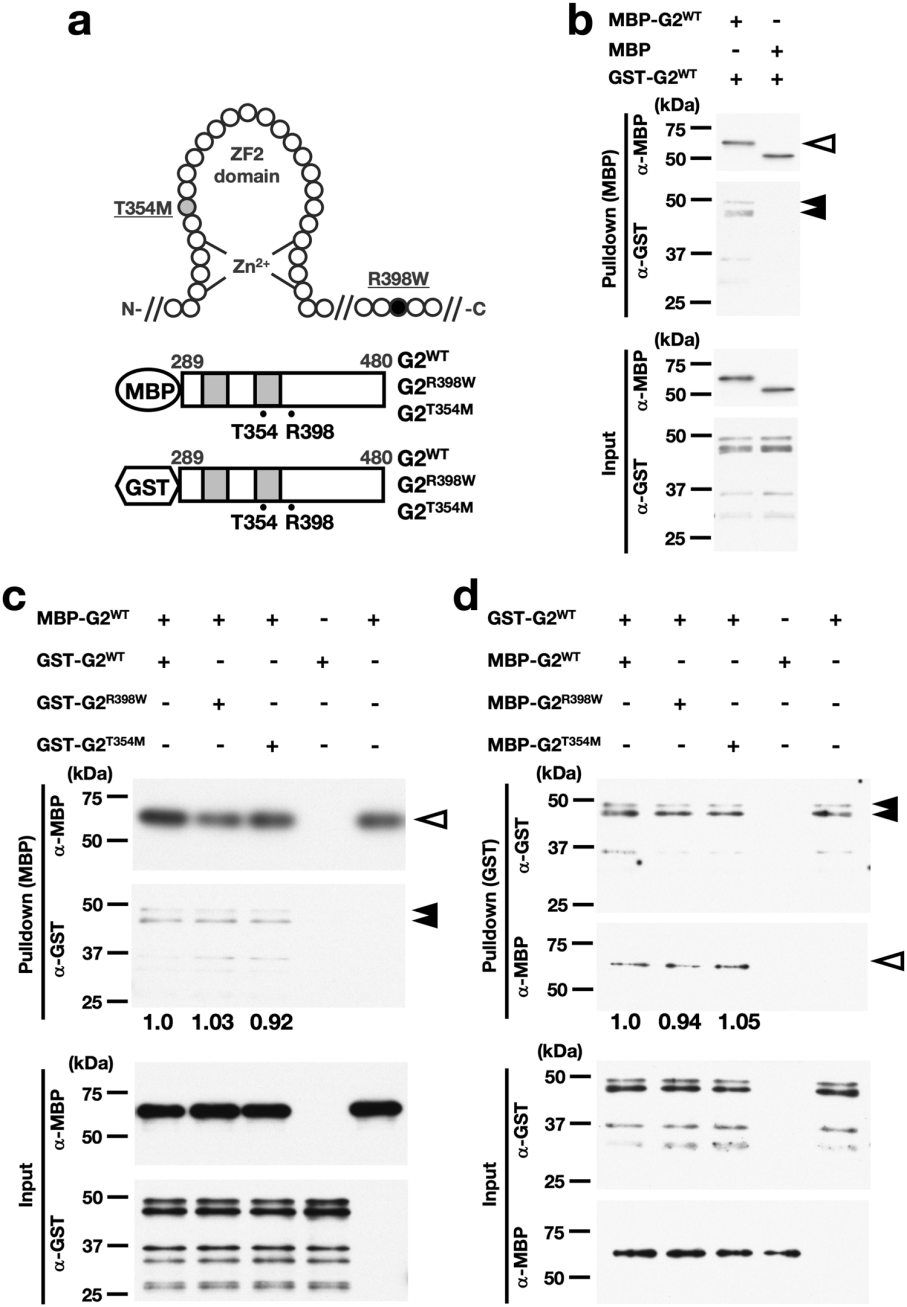
G2^WT^ forms dimers with G2^R398W^ and G2^T354M^ as well as with G2^WT^. **a** Schematic illustration of recombinant MBP-fused and GST-fused GATA2 proteins and the positions of the substituted amino acids. **b**–**d** Pull-down assays of MBP-fused (**b**, **c**) and GST-fused (**d**) GATA2. The indicated recombinant proteins were mixed. MBP- and GST-fused GATA2 were detected by the corresponding antibodies. White and black arrowheads indicate the pull-down signals of MBP-fused and GST-fused GATA2, respectively. Quantification of band intensities in Western blots was performed by the ratio of the GST (**c**) or MBP (**d**) pull-down signal to the input signal. The value in the left-most lane was set to 1.0. Experiments were performed in duplicate, and the results were reproducible.

The DNA-binding activity and transcriptional activity of G2^R398W^ have been reported to be significantly reduced^31^. Therefore, we initially assumed that the mutated *G2*^R398W^ allele would be equivalent to a null allele, but the comparison of the loss-of-function phenotypes in the previous subsection revealed that the *G2*^R398W/+^ mice showed specific phenotypes that are not found in the *G2*^Gfp/+^ mice (Fig. 5), suggesting that the heterozygous expression of the G2^R398W^ protein may interfere with the functions of the intact GATA2 protein by a physical interaction. In this regard, it is interesting to note that two GATA1 molecules form a functional homodimer via reciprocal interactions of the CF and NF domains of each monomer^37–39^. Similarly, GATA3 also forms a homodimer, but in this case, the CF domains interact with each other^40^. Therefore, first performed a pull-down assay using maltose-binding protein (MBP)- and glutathione S-transferase (GST)-fused recombinant proteins covering the GATA2 finger region containing the NF and CF domains (from 289 aa to 480 aa) (Fig. 6a). We found that GST-fused G2^WT^ was successfully pulled down by MBP-fused G2^WT^ but not by MBP alone (Fig. 6b and Supplementary Fig. 5a), indicating that mutant and wild-type GATA2 physically interact with each other and form a homodimer.

To verify whether the R398W and T354M mutations affect the association of GATA2, we constructed GST-fused and MBP-fused G2^R398W^ and G2^T354M^ (Fig. 6a). G2^T354M^ is another germline GATA2 mutation found in patients with DCML deficiency and MonoMAC syndrome patients^29^. We confirmed that both mutant proteins were pulled down by MBP-fused G2^WT^, and the efficiencies were comparable between G2^WT^ and these two mutant proteins (Fig. 6c and Supplementary Fig. 5b). We further examined whether MBP-fused G2^R398W^ and G2^T354M^ could be pulled down by GST-fused G2^WT^. The results clearly demonstrated that the G2^R398W^ and G2^T354M^ proteins were both pulled down by GST-fused G2^WT^ (Fig. 6d and Supplementary Fig. 5c), as was the case for the MBP-fused G2^WT^ experiments. These results thus indicate that the G2^R398W^ and G2^T354M^ proteins have the capacity to dimerize with the G2^WT^ protein and that the homodimerization of G2^R398W^ and G2^WT^ may underlie the pathological basis of the observed phenotypes of the *G2*^R398W/+^ mice.

### Both G2^R398W^ and G2^T354M^ impair the binding of G2^WT^ to the tandem GATA motif

The GATA1 monomer or single GATA1 molecule monovalently binds to the single GATA motif, while the GATA1 homodimer binds bivalently to the tandem GATA motif in which two GATA-binding motifs align in a direct-repeat arrangement using the CF domains of each molecule^39^. Since the homodimerization ability of GATA2 was not changed by the R398W and T354M substitution mutations, we examined whether the DNA-binding ability of G2^R398W^ and G2^T354M^ to the single GATA motif and/or G2^R398W^-G2^WT^ and G2^T354M^-G2^WT^ heterodimers to the tandem GATA motif was affected.

To this end, we prepared the recombinant proteins G2^WT^, G2^R398W^, and G2^T354M^ and conducted surface plasmon resonance (SPR) analyses of the G2^R398W^ and G2^T354M^ monomers binding to the single GATA motif and G2^R398W^-G2^WT^ and G2^T354M^-G2^WT^ heterodimers binding to the tandem GATA motif.

**Fig. 7:**
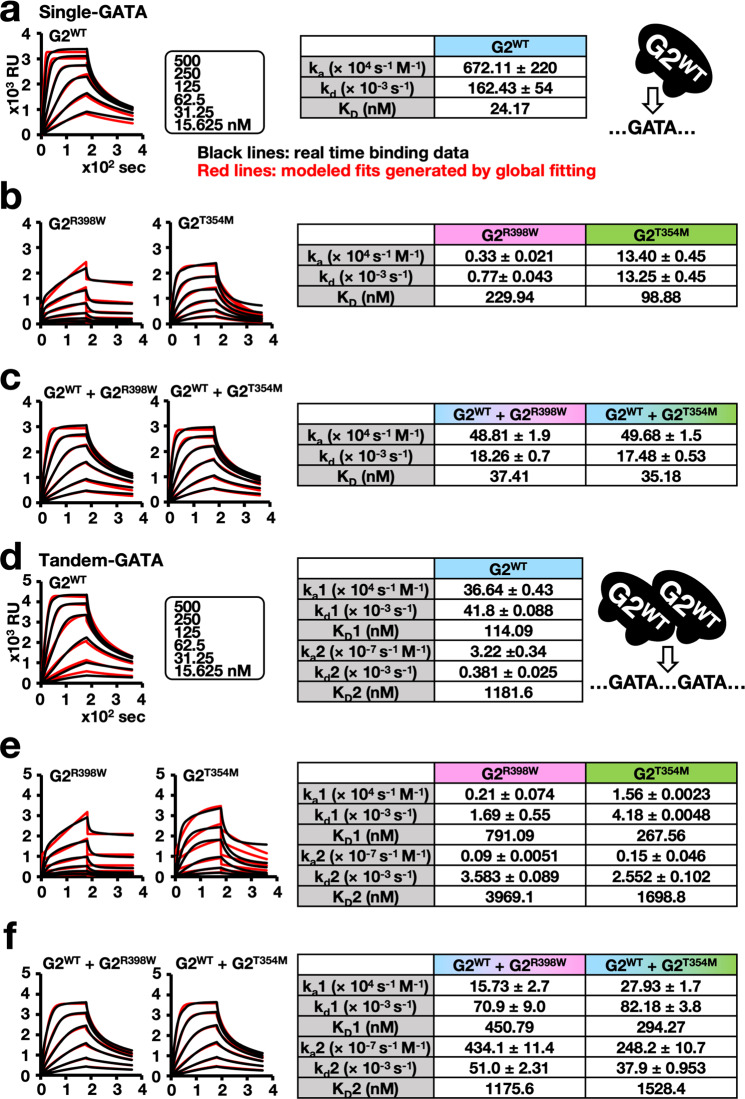
G2^R398W^ and G2^T354M^ disturb G2^WT^ binding to the tandem GATA motif. SPR analyses of GATA2 binding to single (**a**–**c**) and tandem (**d**–**f**) GATA motifs. Schematic diagrams of the binding model of G2^WT^ to single and tandem GATA motifs are shown on the right in **a**, **d**, respectively. The influences of the flow rate of the respective GATA2 proteins at the indicated protein concentrations are shown on the left. Kinetic parameters of the respective GATA2 proteins are shown in the tables. A mixture of equal amounts of the G2^WT^ and G2^mutant^ proteins was used in **c**, **f**. Experiments were performed in duplicate, and the results were reproducible.

As outlined in Fig. 7a, we first examined the binding of the G2^WT^ monomer to a 21-mer DNA containing the single GATA motif. We found that the sensorgram curve of G2^WT^ monomer binding to the single GATA motif showed a very rapid increase, and the binding reached equilibrium within a short time depending on the protein concentrations. In contrast, the binding of G2^R398W^ and G2^T354M^ was weak and took time to reach equilibrium under the conditions tested (Fig. 7b). The dissociation constant (*K*_D_) values of G2^R398W^ and G2^T354M^ were approximately 10-fold and 4-fold lower than those of G2^WT^, respectively (Fig. 7b). These results indicate that the DNA-binding strength of G2^R398W^ and G2^T354M^ was significantly affected by the substitution mutations.

We then analyzed the binding of a 1:1 mixture of G2^WT^:G2^R398W^ and G2^WT^:G2^T354M^ to oligonucleotides containing the single GATA motif. We found that although the *k*_a_ values of the mixture were reduced by one order compared to those of G2^WT^ alone, high-quality sensorgrams were obtained (Fig. 7c). Furthermore, the fit of the data was well correlated with the monovalent binding model. We also found that the *k*_d_ values of the mixtures were reduced by one order compared to that of G2^WT^ alone; consequently, the *K*_D_ values were almost equivalent or within a less than twofold difference between G2^WT^ alone and the mixtures of G2^WT^:G2^R398W^ and G2^WT^:G2^T354M^ (Fig. 7c). This indicates that wild-type GATA2 shows a comparable affinity for the single GATA motif regardless of the coexistence of mutant GATA2.

We next performed SPR analysis using 21-mer DNA containing the tandem GATA motif. We found that the obtained parameters for G2^WT^ were not well fitted to the monovalent binding model. The parameters predicted that the binding fit the two-phase interactions (Fig. 7d). These results indicate that G2^WT^ binds to the tandem GATA motif in a bivalent manner, similar to the case of GATA1^39^.

Compared to the binding of G2^WT^, the binding of G2^R398W^ and G2^T354M^ was weak, and it took time to reach equilibrium under the conditions tested (Fig. 7e). The *K*_D_ values of G2^R398W^ and G2^T354M^ were approximately 8-fold and 2.5-fold lower than those of G2^WT^, respectively (Fig. 7e). These results indicate that the DNA-binding strength of G2^R398W^ and G2^T354M^ to the tandem GATA motif was significantly affected by the substitution mutations. These changes were similar to those observed in the case of the single GATA motif.

Most importantly, when we used a 1:1 mixture of G2^WT^:G2^R398W^ and G2^WT^:G2^T354M^, 2.3- and 1.3-fold decreases in the *k*_a_ values of the first phase of interaction (*k*_a_1) and 1.7- and 2-fold increases in k_d_ values of the first phase of interaction (*k*_d_1) were observed, respectively. Compared to that of the G2^WT^ homodimer, these alterations in the K_D_ values of the first phase of interaction (*K*_D_1) were increased by 4- and 3-fold, respectively (Fig. 7f). These decreases in binding affinity for the tandem GATA motif stood in clear contrast to the situation for the single GATA motif. These results thus support our hypothesis that the heterodimer of the G2^mutant^ (G2^R398W^ or G2^T354M^) markedly lost its DNA-binding ability. The coexistence of G2^R398W^ or G2^T354M^ with G2^WT^ in the heterodimer seems to strongly disturb the ability of the G2^WT^:G2^mutant^ heterodimer to bind to the tandem GATA motif.

### The G2^WT^:G2^mutant^ heterodimer loses its transactivation activity in the tandem GATA motif

To evaluate how the coexistence of the GATA2 mutant in the heterodimer affects the transcriptional activity of the G2^WT^:G2^mutant^ heterodimer, we conducted luciferase reporter assays by using HEK293T cells. In particular, we focused on the transcriptional activity of GATA2 via the tandem GATA motif. For this purpose, we constructed three luciferase reporters containing single or two types of tandem GATA motifs (AGATAAGATAA-type and AGATAAAGATAA-type) placed in triplicate on the upstream side of the minimal promoter-driven *Luc2CP* gene and performed transient transfection assays.

**Fig. 8:**
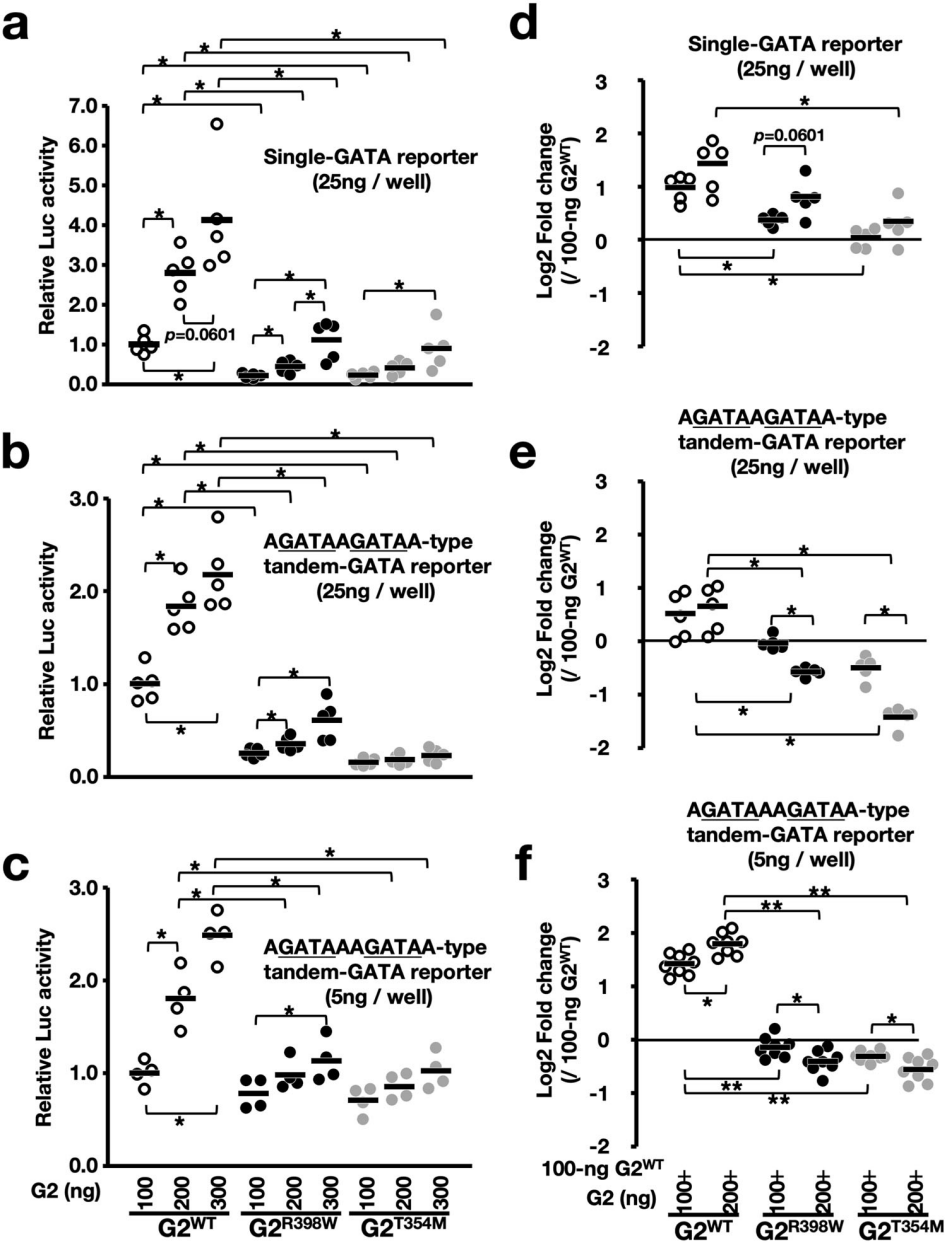
Dominant-negative effect of G2^R398W^ and G2^T354M^ on transactivation activity. **a**–**c** Scatter dot plots of the transcriptional activity measured by luciferase reporter assay in HEK293T cells following transfection with luciferase reporter constructs containing single (**a**) and tandem (**b**, **c**) GATA motifs together with the respective GATA2 expression constructs at doses of 100, 200, and 300 ng/well. AGATAAGATAA-type (middle) and AGATAAAGATAA-type (bottom) tandem GATA constructs were used for transient reporter/effector cotransfection assays. Upper and middle panels, *n* = 5 biologically independent samples and bottom panel, *n* = 4. **d**–**f** Similar sets of experiments in which 100 ng of the G2^WT^ expression construct and luciferase reporter construct containing single (**d**) and tandem (**e**, **f**) GATA motifs were concomitantly transfected with G2^WT^, G2^R398W^, or G2^T354M^ expression constructs at doses of 0, 100, and 200 ng/well. AGATAAGATAA-type (middle) and AGATAAAGATAA-type (bottom) tandem GATA constructs were used for transient reporter/effector cotransfection assays. Upper and middle panels, *n* = 5 biologically independent samples and bottom panel, *n* = 8. The average luciferase activity of G2^WT^ at a dose of 100 ng/well was set to 1.0 in **a**–**c**, and fold changes of the average value of 100 ng of G2^WT^ are shown in **d**–**f**. *p < 0.05.

When transfected individually with the single GATA and two types of tandem GATA reporters, the transactivation activities of G2^R398W^ and G2^T354M^ were significantly reduced compared with that of G2^WT^, regardless of the configuration of GATA-binding motifs (Fig. 8a–c). It is worth mentioning that the transactivation activities of G2^R398W^ and G2^T354M^ in the single GATA motif were increased, albeit slightly, in a dose-dependent manner, and the activity at a dose of 300 ng became equivalent to that of G2^WT^ at a dose of 100 ng (Fig. 8a). In contrast, a 3-fold increase in the transfected constructs of G2^R398W^ and G2^T354M^ could not compensate for the decrease in transactivation activity in the tandem GATA motif (Fig. 8b, c). Thus, while functional defects caused by structural mutations can be partially compensated by an increase in quantity, the transactivation activity in the tandem GATA motif requires many more constructs than the single GATA motif.

To elucidate how coexpression of G2^R398W^ and G2^T354M^ influences the transactivation activity of G2^WT^, we performed a transfection assay again by using 100 ng of G2^WT^ as the baseline amount. We then added an equal or twofold higher amount of either G2^WT^, G2^R398W^, or G2^T354M^ to the basal 100 ng of G2^WT^. When we used the single GATA motif reporter, the luciferase activity was increased in a dose-dependent manner in all G2^WT^, G2^R398W^, and G2^T354M^ cases (Fig. 8d). In this assay, the reporter activity at the basal 100-ng G2^WT^ addition was set as 0. Compared with the incremental increase in luciferase reporter activity with the addition of the G2^WT^ expression plasmid, the effects of G2^R398W^ and G2^T354M^ additions were fairly small.

In contrast, when we used the tandem GATA motif reporters, the addition of G2^R398W^ and G2^T354M^ to G2^WT^ showed reverse dose-dependent effects against the transactivation activity of G2^WT^. While the addition of G2^WT^ caused an increase in transactivation activity in a dose-dependent manner, both GATA2 mutants appeared to repress their transactivation activities at two conditions, i.e., with 25-ng AGATAGATAA-type tandem-GATA reporter (Fig. 8e) and 5-ng AGATAAGATAA-type tandem-GATA reporter (Fig. 8f). G2^T354M^ worked as a less effective activator of the single GATA motif than G2^R398W^, but it worked as a more effective repressor of the tandem GATA motifs. Interesting observation here is that, effect size of GATA2 mutants on AGATAAGATAA-type tandem-GATA was smaller than that on AGATAGATAA-type tandem-GATA (Fig. 8e, f). Indeed, when we used 25-ng AGATAAGATAA-type tandem-GATA reporter, the dose-dependent adverse effect by coexisting G2^R398W^ on the transactivation activity of G2^WT^ was modest and not so significant (Supplementary Fig. 6). We speculate that, when effector/reporter ratio is low in a transient reporter assay, a single GATA2 molecule is prone to bind to the tandem GATA motif, especially in case of tandem GATA motif with multiple spacer nucleotides, while GATA2 dimers are prone to be produced and bind to the tandem GATA motif when the concentration of GATA2 molecules becomes high. Thus, leverage over the coexisting G2^WT^ varied in accordance with the nature of the mutant GATA2, the configuration of GATA-binding motifs and probably with the expression level of GATA2 during hematopoiesis.

To further examine the negative effect of the GATA2 mutant on the transactivation activity of wild-type GATA2 from the chromatin integrated reporter, we prepared two types of cells stably carrying the reporter constructs of single or AGATAAGATAA-type tandem GATA motif and introduced GATA2 expression plasmids by episomal transfection. Showing strong agreement with the results of transient transfection reporter assays, the expression of G2^R398W^ and G2^T354M^ repressed the transcriptional activity of G2^WT^ in a dose-dependent manner in the genome-integrated tandem motif but not in the single motif (Supplementary Fig. 7). These results further support the conclusion that these GATA2 mutants repress wild-type GATA2 activity in the heterodimer.

### Fluctuating expression of genes in *G2*^R398W/+^ LSKs

**Fig. 9:**
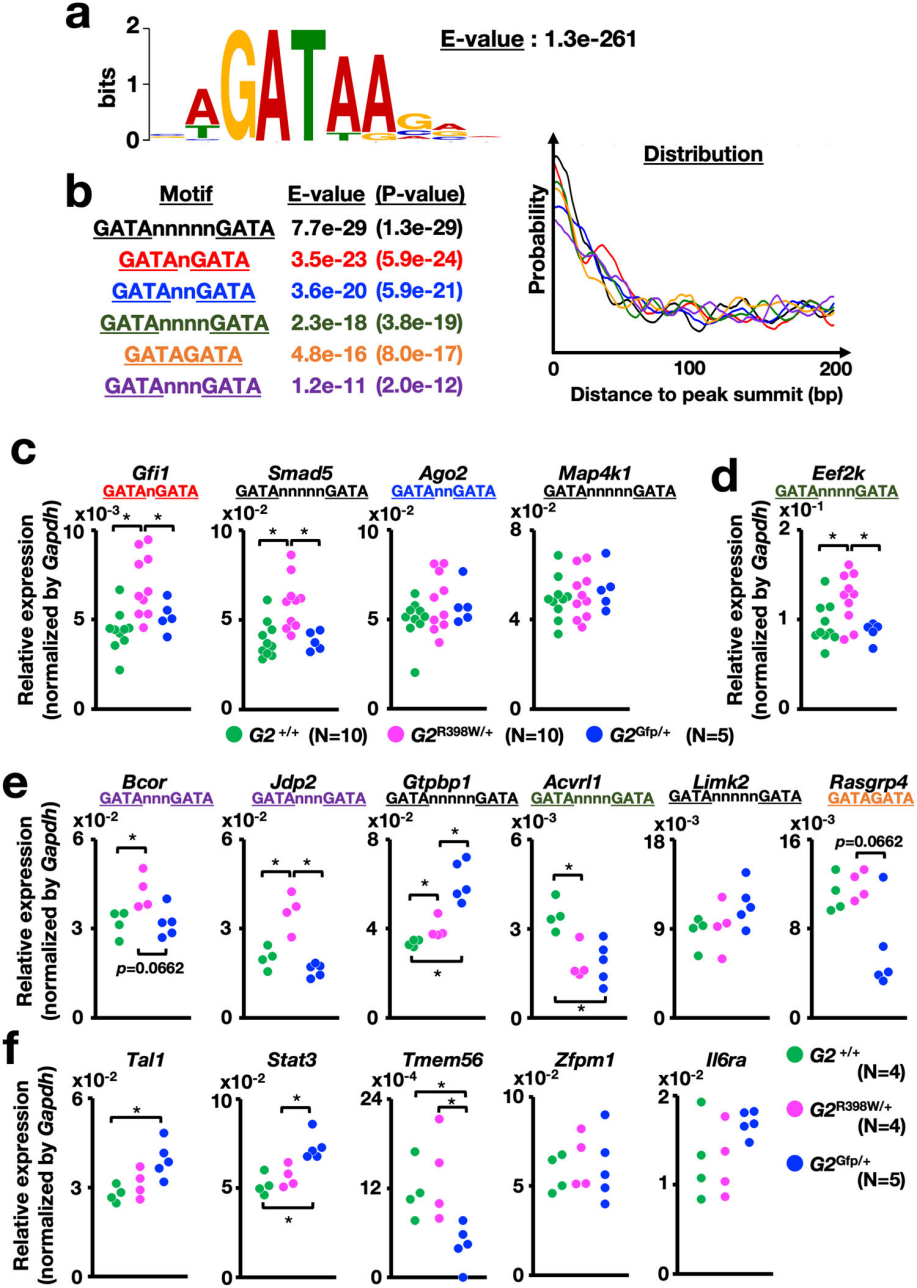
Expression landscape of genes containing tandem GATA motifs in ChIP-Seq peak regions. **a** GATA2-binding motif in ChIP-Seq peak regions in the GATA2 ChIP-Seq dataset of murine bone marrow lineage-negative cells. **b** Tandem GATA motifs were used to search ChIP-Seq peak regions containing tandem GATA motifs. Motifs are arranged in ascending order according to the E-value. The right panel shows the distance distributions between ChIP-Seq peak summits and the center of the motifs with color-matched lines. **c**–**e** Dot plots for the expression of genes containing the tandem GATA motif(s) in bone marrow LSK cells of 5- to 6-month-old mice. Genes commonly found in two datasets (**c**), found in the ENCODE dataset (**d**), and found in the ChIP-Atlas dataset (**e**) are shown. Tandem GATA motif types found in the ChIP-seq peaks in individual genes are shown. **f** Dot plots for the expression of genes harboring only a single GATA motif(s) in their proximity in bone marrow LSK cells of 5- to 6-month-old mice. *p < 0.05. Numbers of mice are indicated in the figure.

We next verified whether coexpressing G2^R398W^ altered the expression of GATA2 target genes that contain the tandem GATA motif in the gene loci. To this end, we analyzed a GATA2 chromatin immunoprecipitation sequencing (ChIP-Seq) dataset of murine bone marrow lineage-negative cells obtained from the ChIP-Atlas database. A consensus GATA-binding motif was enriched in the peaks of 2193 ChIP-Seq peaks with a high enrichment value (E-value; Fig. 9a). These ChIP-Seq peak loci contain several types of tandem GATA motifs that contain various lengths of spacers (*n* = 0–5). In Fig. 9b, the identified tandem GATA motifs were aligned by using the E-values. Through this inspection of the 2193 ChIP-Seq peaks, we extracted 314 peaks that contained at least one tandem GATA motif with significant enrichment. We identified 103 genes that harbor tandem GATA motif(s) within ±100 kbp of the transcription start site (TSS) (Supplementary Table 1).

We further analyzed the ENCODE GATA2 ChIP-Seq dataset of G1E cells^41^ and found 39 genes containing tandem GATA motif(s) (Supplementary Table 1). Ten genes overlapped in two different datasets.

We then performed qRT-PCR analysis and confirmed the expression of 4 genes out of 10 in bone marrow LSK cells. In particular, the expression of *Gfi1* and *Smad5*, both of which have been shown to be important hematopoietic regulators^42,43^, was significantly increased in the *G2*^R398W/+^ mice but not in the *G2*^Gfp/+^ mice (Fig. 9c). In addition, we chose the *Eef2k* gene as a tandem GATA motif-containing gene from the ENCODE dataset since Eef2k is known to regulate HSC apoptosis^44^ and found changes in expression in *G2*^R398W/+^ mice (Fig. 9d). Furthermore, we chose the *Bcor*, *Jdp2, Gtpbp1, Acvrl1, Limk2,* and *Rasgrp4* genes from the ChIP-Atlas database based on their involvement in hematopoiesis and/or leukemogenesis^45–50^ and found that the expression of the *Bcor* and *Jdp2* genes was significantly elevated only in the *G2*^R398W/+^ mice but not in the *G2*^Gfp/+^ mice (Fig. 9e). Genes whose expression was changed in both *G2*^R398W/+^ and *G2*^Gfp/+^ mice or only in *G2*^Gfp/+^ mice were also identified (Fig. 9e).

We next analyzed the expression of genes that have a single GATA motif but without any tandem GATA motifs in proximity. We chose the *Tal1*, *Stat3*, *Tmem56*, *Zfpm1,* and *Il6ra* genes, since they were reported as downstream genes of GATA2^3,30,36,51,52^. While we could not find any genes whose expression was changed solely in the *G2*^R398W/+^ mice, we could identify genes changed only in *G2*^Gfp/+^ mice (Fig. 9f). Taken together, our data suggest that heterozygous expression of G2^R398W^ affects GATA2 regulatory effects through highly inactive heterodimer formation, which preferentially affects the tandem GATA motif-mediated regulation of GATA2.

## Discussion

Orchestrated and elaborate regulation of hematopoiesis-associated genes is critical for the maintenance of hematopoietic homeostasis, and it has been shown that the transcription factor GATA2 plays a central role in the maintenance of hematopoietic homeostasis^14–20,34^. Recent advances in clinical research have led to an emerging theory that the *GATA2* gene is causative for congenital hematopoietic diseases in autosomal dominant traits, including Emberger syndrome, DCML deficiency, and MonoMAC syndrome. However, since simple heterozygous *Gata2*-knockout mice did not show phenotypes recapitulating these human diseases^16,34^, we surmise that the pathogenesis of GATA2-related immunodeficiency disease includes mechanisms much more complex than a simple quantitative deficit. To address such mechanisms, it seems necessary to develop more elaborate mouse models. To this end, we generated mouse lines carrying a heterozygous single-nucleotide substitution mutation (c.1192C>T, p.398Arg>Trp) in the *Gata2* gene by a genome editing strategy in this study. This mutation was found in a family with DCML deficiency. We found that, in contrast to the heterozygous GATA2-null (*G2*^–/+^) mutant mice, the *G2*^R398W/+^ mice had significantly decreased numbers of BCs, NKCs, mDCs/pDCs and monocytes, but the numbers of both CD4^+^ TCs and CD8^+^ TCs as well as granulocytes were comparable to those of the control mice. Thus, the phenotypes of the *G2*^R398W/+^ mice nicely recapitulate the clinical observations of the diseases caused by the heterozygous GATA2 mutation, demonstrating that the *G2*^R398W/+^ mice serve as an excellent disease model mouse line.

**Fig. 10:**
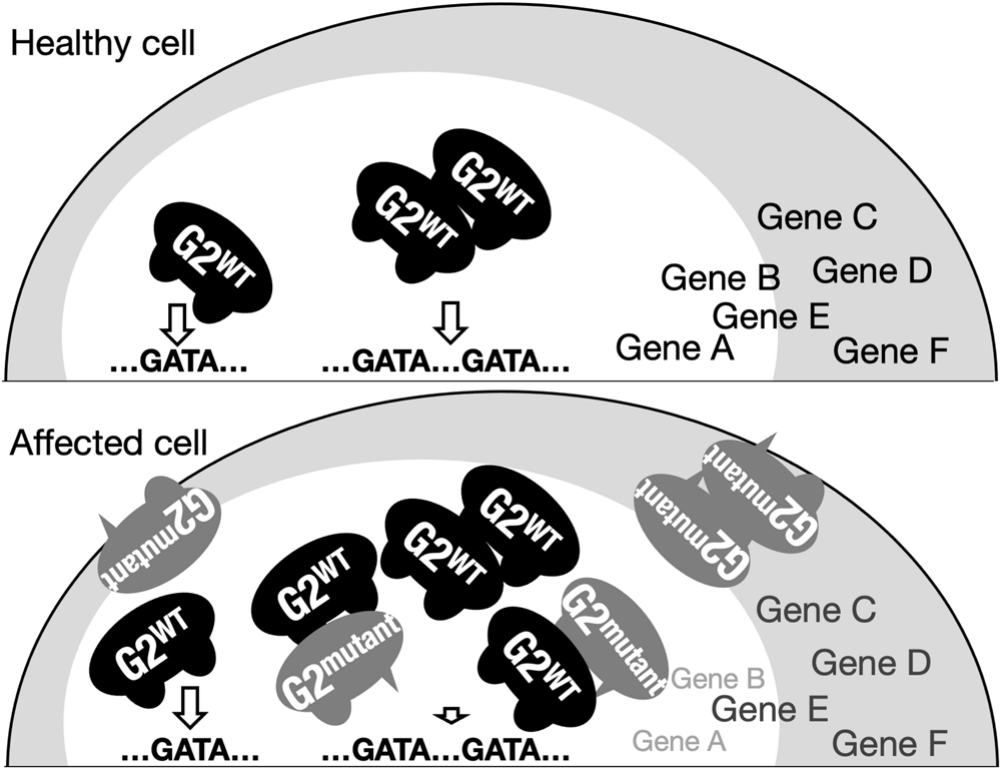
A model for the pathogenesis of GATA2-related diseases caused by structural mutation. G2^WT^ and G2^mutant^ coexist in cells of affected individuals carrying heterozygous *GATA2* gene mutations that produce GATA2 structural mutants. In cases of the G2^mutant^ with impaired DNA-binding activity to the CF domain, the G2^mutant^ effectively disrupts the function of G2^WT^ on the tandem GATA motif and is barely involved in the function of G2^WT^ on the single GATA motif. Genes whose regulation is heavily dependent on the tandem GATA motif are predicted to be subject to strong interference by the presence of the G2^mutant^.

Mutations found in GATA2-related diseases are divided into two categories^29^. One is quantitative deficits of the GATA2 protein, while the other is qualitative defects of the GATA2 protein. In the former case, a massive reduction in *GATA2* gene expression usually occurs, and only a low level of the GATA2 protein is expected to reside in the cells, suggesting that a reduced amount of intact GATA2 protein is involved in the pathogenesis of these diseases. In contrast, in the latter cases, the mutated alleles produce structural mutants of GATA2 that coexist with intact GATA2 from the other wild-type allele. Most of the mutations in this category are concentrated in the CF domain of GATA2^29^, severely impairing the DNA-binding activity of GATA2^31,53^. As summarized in Fig. 10, we demonstrated in this study that two of the latter GATA2 mutants (p.398Arg>Trp and p.354Thr>Met) harbor weak binding activity to single and tandem GATA motifs. However, the coexistence of these GATA2 mutants significantly affected the function of coexisting wild-type GATA2 specifically through tandem GATA motifs, although the effect size of GATA2 mutants varied in accordance with the nature of tandem GATA motif, whereas the pristine function of wild-type GATA2 through the single GATA motif was not substantially altered by the coexistence of the GATA2 mutant. Based on these observations, we propose that the expression of GATA2 target genes, which is heavily dependent on the tandem GATA motif, is markedly influenced by the presence of heterozygous missense mutations, which results in the disorganized regulation of GATA2 and disruption of hematopoietic homeostasis, leading to the onset of DCML deficiency.

We identified several genes that showed significant differences in expression levels between *G2*^R398W/+^ and *G2*^+/+^ mice through our qRT-PCR analysis coupled with ChIP-Seq database analysis. The expression of these genes was not changed between *G2*^Gfp/+^ and *G2*^+/+^ mice. These results suggest that the products of those genes may be responsible for the phenotypes of the qualitative defect of GATA2. In particular, *Smad5* has been shown to be a GATA2-target gene and a key regulator of BMP signaling^52,54,55^, which supports HSC heterogeneity and lineage output decisions in the myeloid and lymphoid axes^56^. We envisage that perturbations in the expression of these GATA2 target genes may disturb the proliferation and differentiation of hematopoietic lineage cells.

We generated compound heterozygous mutant mice (*G2*^R398W/Gfp^) utilizing mice harboring the *G2*^Gfp^ knockin-knockout allele^12^ and *G2*^R398W^ allele. This *G2*^R398W/Gfp^ mouse carries *G2*^R398W^ and *G2*^Gfp^ alleles so that GATA2 from the single G2^R398W^ allele supports the function of GATA2 in the mice. We found that the *G2*^R398W/Gfp^ mice survived at E10.5, which was beyond the critical lethal point of complete GATA2-knockout embryos^11^. We also found that *G2*^R398W^ homozygotes (*G2*^R398W/R398W^) survived beyond E12.5, which is markedly longer than *G2*^R398W/Gfp^ mice. However, the *G2*^R398W/R398W^ embryos are not alive at the time of birth. In a reporter cotransfection-transactivation assay, we showed that G2^R398W^ retains some extent of its transactivation activity. We surmise that the residual transcriptional activity of G2^R398W^ contributes to the prolonged survival of *G2*^R398W/R398W^ and *G2*^R398W/Gfp^ embryos beyond the critical period in an allele number-dependent manner.

The *G2*^R398W/+^ mice showed a phenotype recapitulating that of DCML deficiency, but the mice never developed hematopoietic neoplasms. In contrast, mice heterozygous for the *Gata2*-null mutation (*G2*^-/+^) did not show an obvious phenotype except for a large number of defective hematopoietic stem and progenitor cells^16,34^. The expression of the *Gata2* gene in the LSK cells of *G2*^-/+^ mice was reduced by approximately 50% of the wild-type level^34^ contrast, *G2*^fGN/fGN^ mice that harbor approximately 20% of the GATA2 expression of wild-type mice are prone to develop progressive myelomonocytosis resembling CMML without leukocytopenia^36^. These observations revealed that the 20% expression level of GATA2 in the mice cannot fully explain human GATA2-related diseases. While quantitative deficits of GATA2 are indeed involved in the pathogenesis of MPNs, this study unequivocally demonstrates that the GATA2-related diseases provoked by heterozygous substitution mutations utilize specific mechanisms linked to qualitative deficits.

In conclusion, we identified in this study that mice harboring a heterozygous GATA2 mutant with weakened DNA-binding activity develop multilineage cytopenia of BCs, NKCs, mDCs, pDCs, and monocytes due to interference with coexisting wild-type GATA2 activity in a GATA motif configuration-dependent manner. Mutant GATA2 interferes with wild-type GATA2 activity via a dominant-negative mechanism. While in human patients, GATA2 structural mutations were scattered over a wide area of the GATA2 protein, we believe that the mechanism clarified in this study explains one type of GATA2-related disease. Thus, our *G2*^R398W/+^ mice will serve as a unique and vital model to study qualitative deficits of GATA2.

## Methods

### Animal studies

Experimental procedures for animals were approved by the Institutional Animal Experiment Committee of Tohoku University. Experiments were carried out in accordance with the Regulation for Animal Experiments at Tohoku University. The plasmid vector pX330 expressing Cas9 and gRNA was digested with BbsI, and a pair of DNA oligos recognizing the *Gata2* target site (5′-AGG GAT CCA GAC CCG GAA T −3′) was ligated to the linearized vector. Donor DNA oligos encoding an arginine to tryptophan substitution were designed as follows: 5′-ACC ATG AAG AAG GAA GGG ATC CAG ACC CGG AAT TGG AAG ATG TCC AGC AAA TCC AAG AAG AGC AAG AAA G-3′ (the nucleotide corresponding to the c.1192 C > T substitution is underlined). The plasmid and donor DNA were coinjected into BDF1-fertilized eggs. The founder mice were crossed with C57BL/6 J mice for at least 6 generations, and progeny were used in this study. Primer sequences for genotyping PCR are listed in Supplementary Table 2. Images of embryos and fetal livers were captured with an MZFL III stereomicroscope (Leica) and a DP73 CCD camera (Olympus) using the manufacturer’s standard software. Blood samples were obtained from the retro-orbital plexuses of mutant mice and their unaffected littermates, and hematopoietic indices were measured with a Celltac-α autohemocytometer (Nihon Koden).

### Flow cytometry analysis

Whole blood was hemolyzed with ammonium chloride hemolytic buffer and washed twice with phosphate-buffered saline. Lineage depletion of bone marrow cells was performed using a cocktail of biotinylated antibodies against Ter119, B220, Gr1, and CD8 (purchased from BioLegend) and CD4, CD11b and CD127 (eBioscience), followed by removal using Dynabeads M-280 streptavidin-conjugated magnetic beads (Thermo Fisher Scientific). The combinations of fluorescently labeled antibodies used in the comparison between *G2*^R398W/+^ and *G2*^Gfp/+^ mice with *G2*^+/+^ mice are shown in Supplementary Tables 3 and 4, respectively. The stained cells were analyzed and sorted by means of BD FACSAria II and BD FACSDiva software (Becton Dickinson). BCs, NKCs, mDCs, pDCs, monocytes, CD4^+^ TCs, and CD8^+^ TCs and granulocytes were defined as B220^+^CD19^+^CD4^–^CD8^–^, CD49b^+^NK1.1^+^NKG2D^+^CD11b^+^CD1d^–^CD3^–^, CD11b^+^CD11c^+^F4/80^–^, CD11b^–^CD11c^+^F4/80^–^Gr1^+^, CD11b^+^CD11c^–^F4/80^+^Gr1^–^, B220^–^CD19^–^CD4^+^CD8^–^, and B220^–^CD19^–^CD4^–^CD8^+^, respectively. Absolute numbers of each cell subset were calculated by the following formula: absolute count/μl = WBC count (/μl) x percentage of cells in total mononuclear cells determined by flow cytometry. Long-term (LT)-HSCs, ST-HSCs, MPP2s, MPP3s, and MPP4s were defined as CD150^+^Flk2^–^CD48^–^, CD150^–^Flk2^–^CD48^–^, CD150^+^Flk2^–^CD48^+^, CD150^–^Flk2^–^CD48^+^, and CD150^–^Flk2^+^CD48^+^, respectively, in the LSK-gated fraction^57^. Occasionally, LSK cells were subfractionated based on the expression of CD150 and CD48 into CD150^+^CD48^–^LSKs (HSCs), CD150^–^CD48^–^LSKs (MPPs), and CD150^–^CD48^+^LSKs (restricted progenitors)^58^. CMPs, GMPs, and MEPs were defined as CD34^+^CD16/32^med^, CD34^low/+^CD16/32^high^, and CD34^low^CD16/32^–^, respectively, in the LK-gated fraction^59^.

### Purification of recombinant protein and interaction analysis

MBP-fused and GST-fused murine G2^WT^, G2^R398W^, and G2^T354M^ were produced in *Escherichia coli* BL21(DE3)pLysS-competent cells (Novagen)^60^, and the recombinant proteins were purified by affinity chromatography utilizing a Profinia instrument (Bio-Rad) according to the manufacturer’s protocol. For the pull-down assay, the recombinant MBP-fused proteins and each of the corresponding GST-fused proteins were incubated in binding buffer^60,61^ at room temperature. Protein complexes were purified with amylose magnetic beads (New England Biolabs) or glutathione magnetic beads (Thermo Fisher Scientific) and then detected by immunoblot analysis. SPR analysis was performed with a Biacore-X100 instrument (GE Healthcare). Double-stranded DNA probes in which the 5′ ends of sense strands were biotinylated were purified by native PAGE. Subsequently, each probe was immobilized in one of two streptavidin-coated flow cells in a Sensor chip-SA (GE Healthcare) as an active flow cell of 1000 resonance units (RU) DNA according to the manufacturer’s protocol. The other flow cell was left blank for reference subtraction. Data processing was performed with Biacore-X100 evaluation software (GE Healthcare). The sequences of the DNA probes were 5′-GCGCTCAGAGATAAGGCCTTG-3′ and 5′-GCGAGATAAGATAAGGCCTTG-3′.

### Luciferase reporter analysis

For transient reporter/effector cotransfection assays, single GATA and two types of tandem GATA reporter constructs were generated by inserting fragments of TCCGGCAACAGATAAGGAATCCCTG-3′, 5′-TCCGGCAACAGATAAGATAAGGAATCCCTG-3′ or 5′-TCCGGCAACAGATAAAGATAAGGAATCCCTG-3′ in triplicate into the multiple cloning site of pGL4.28 vector (Promega). HEK293T cells (ATCC CRL-3216) were washed twice with phosphate-buffered saline (PBS) and resuspended in 500 μl of fresh medium at the concentration of 1.5 × 10^5^/ml. Effector plasmids containing EF1α-promoter-driven murine G2^WT^ and/or G2^R398W^ or G2^T354M^ expression vectors were adjusted in total amount to 300 ng with empty vector and transfected into HEK293T cells with reporter constructs. 25 ng of single GATA, 25 ng of AGATAAGATAA-type tandem GATA, and 5 and 25 ng of AGATAAAGATAA-type tandem GATA constructs were used for transient reporter/effector cotransfection assays. 0.75 ng of *Renilla Luc* expression vector was included in the transfections as an internal control. After 24 h of transfection, luciferase activity was measured at each dosage point of effector expression vectors with the Dual-Luciferase reporter assay system (Promega) with Lumat LB 9507 (Berthold Technologies) according to the manufacturer’s protocol. Data are expressed as the mean of duplicate or triplicate well from ≥4 independent experiments. For transient transfection assays, single GATA and tandem GATA reporter constructs were generated by inserting fragments of 5′-TCCGGCAACAGATAAGGAATCCCTG-3′ or 5′-TCCGGCAACAGATAAGATAAGGAATCCCTG-3′ in triplicate into the multiple cloning site of pNL1.3 reporter vector (Promega). HEK293T reporter cell lines carrying the single GATA or AGATAAGATAA-type tandem GATA reporter construct were established. Cells were washed twice with PBS and resuspended in 500 μl of fresh medium at the concentration of 1.5 × 10^5^/ml followed by transfection with G2^WT^ and/or G2^R398W^ or G2^T354M^ expression vector at the indicated dosages. The total amount of expression vector was adjusted to 300 ng with empty vector. Five microliters of culture medium was collected 24 h after transfection and used for measurement of luciferase activity with the Nano-Glo luciferase assay system (Promega) with the PHERAstar multiplate reader (BMG Labtech) according to the manufacturer’s protocol. Luciferase value was defined as the subtraction of luciferase activity measured in the cultured medium of untransfected cells. Date are expressed as the mean of triplicate well from ≥3 independent experiments.

### Immunoblot analysis

Bone marrow cell nuclear extracts and a recombinant protein solution were mixed 1:1 with 2X Laemmli buffer and subjected to SDS-PAGE in a 10% polyacrylamide gel. The Precision Plus Protein Kaleidoscope Standard (Bio-Rad) was loaded into neighboring lanes as a molecular-weight marker. After electrophoresis, the separated proteins were transferred to PVDF membranes. The proteins were probed with primary anti-GATA2 (dilution ratio: 1:5000, Perseus Proteomics), anti-Lamin B (1:5000, Santa Cruz Biotechnology), anti-MBP (1:10,000, Santa Cruz Biotechnology), and anti-GST (1:5000, Santa Cruz Biotechnology) antibodies, followed by detection of the primary antibodies with horseradish peroxidase-conjugated secondary antibodies (1:5000-1:20,000, Invitrogen-Life Technologies). Signals were visualized on X-ray film using ECL-Prime Western blotting Detection Reagents (GE Healthcare) according to the manufacturer’s protocol. Signal values were quantified using ImageJ software.

### Quantitative RT-PCR analysis

RNA was isolated from sorted cells using ISOGEN-LS (NIPPON GENE). Subsequently, first-strand cDNA was synthesized using ReverTra Ace (TOYOBO). Quantitative real-time PCR was carried out with a StepOnePlus Real-Time PCR System (Applied Biosystems) using THUNDERBIRD SYBR qPCR mix (TOYOBO) in triplicate wells for each reaction, and the results of the triplicate wells were averaged. The data were normalized to the expression level of *Gapdh* as an internal control. Primer sequences are described in Supplementary Table 5. Data for which relevant values were obtained from all mice are shown.

### Analysis of GATA2 ChIP-Seq data

The GATA2 ChIP-Seq dataset (SRX035992) in murine bone marrow lineage-negative cells from the ChIP-Atlas database (https://chip-atlas.org/) and dataset (ENCSR000DIE) in the G1E cell line from the ENCODE consortium were analyzed. Motif enrichment analysis and motif searching were performed with the MEME-ChIP and FIMO programs in MEME Suite^62^, respectively. The discovered motifs were mapped to the reference genome using the Genomic Regions Enrichment of Annotations Tool (GREAT)^63^.

### Statistics and reproducibility

In all, 4–10 mice for each genotype (i.e. WT, *G2*^R398W/+^, and *G2*^Gfp/+^) were used for flowcytometry, qRT-PCR, and immunoblot and hematopoietic indices analyses. 13 and 8 *G2*^R398W/+^ females were mated with *G2*^R398W/+^ and *G2*^Gfp/+^ males, respectively, and analyzed the genotypes of pups. Luciferase assays were performed using at least 4 biologically independent samples. Statistical analyses were performed with the Mann–Whitney *U* test. *p*-values were considered significant if *p* < 0.05. The pull-down and SPR analyses were performed in two technical replicates in two independent experiments.

### Reporting summary

Further information on research design is available in the Nature Research Reporting Summary linked to this article.

## Supplementary information

Supplementary Information

Description of Additional Supplementary Files

Supplementary Data 1

Reporting Summary

## Data Availability

Source data underlying Figs. 1d–g, 3a, b, 4a, b, 5a, b, 7a–f, 8a, b, 9c–f, S1a, b, S2a, b, S3a, b, and S4a, b are presented in Supplementary Data 1. Original source data for the Figs. 1c, g, and 6b–d are also presented in Supplementary Data 1. The GATA2 ChIP-Seq datasets in murine bone marrow lineage-negative cells and in the G1E cell line are available from the ChIP-Atlas database (accession code SRX035992) and from the ENCODE consortium (accession code ENCSR000DIE), respectively.
