# Supplementary material for: Heterozygous variants in GATA2 contribute to DCML deficiency in mice by disrupting tandem protein binding
Source: Commun Biol. 2022 Apr 19;5:376. doi: 10.1038/s42003-022-03316-w (PMC9018821; doi:10.1038/s42003-022-03316-w)
Supplement: Supplementary file 1 — Supplementary Information [file 42003_2022_3316_MOESM1_ESM.pdf]

Supplementary Information

Supplementary Figure 1

Uncut blots for Figure 1c and 1g.

Supplementary Figure 2

Evaluation of the peripheral blood counts of *G2*$^{R398W/+}$ mice at 5-7 months old.

Supplementary Figure 3

Evaluation of the peripheral blood counts of 3-month-old *G2*$^{R398W/+}$ mice.

Supplementary Figure 4

Evaluation of the peripheral blood counts of 6-month-old *G2*$^{R398W/+}$ mice in the 2nd line.

Supplementary Figure 5

Uncut blots for Figure 6b, 6c and 6d.

Supplementary Figure 6

Luciferase reporter analysis using 25 ng of AGATAAGATAA-type tandem-GATA reporter.

Supplementary Figure 7

Luciferase reporter analysis using HEK293T cells stably carrying a reporter construct.

Supplementary Table 1

List of genes identified in the GATA2 ChIP-seq datasets, which harbor tandem GATA motifs within ±100 kbp of the TSS.

Supplementary Table 2

Primer sequences for genotyping PCR.

Supplementary Table 3

Combinations of fluorescently labeled antibodies for flow cytometry.

Supplementary Table 4

Combinations of fluorescently labeled antibodies for flow cytometry of cells from *Gfp* knockin mice.

Supplementary Table 5

Primer sequences for quantitative RT-PCR.

Supplementary References

Supplementary Figure 1

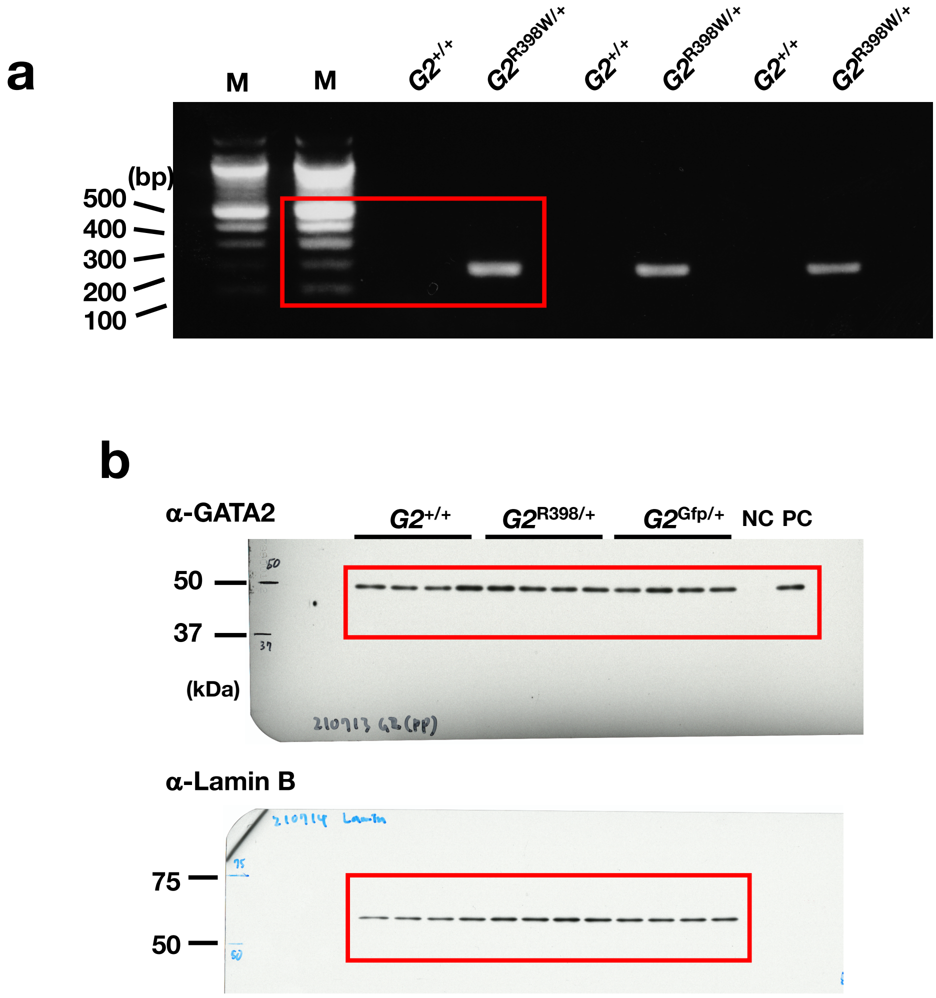

Uncut blots for Figure 1c and 1g.

a Uncut blot for PCR-based genotyping strategy of the *G2*^{R398W} allele. Image used for Figure 1c is shown in red square. **b** Uncut blot for immunoblot analysis of nuclear extracts from bone marrow cells using anti-GATA2 (upper panel) and anti-Lamin B(lower panel) antibodies. Images used for Figure 1g are shown in red squares.

.

Supplementary Figure 2

a

(N = 10)	*G2*$^{+/+}$	*G2*$^{R398W/+}$
WBC (x10^2/μL)	109.6 ± 30.6	65.7 ± 32.1
RBC (x10^4/μL)	993 ± 30	958 ± 33
HGB (g/dL)	13.9 ± 0.6	13.8 ± 0.8
HCT (%)	46.4 ± 1.1	45.4 ± 1.1
MCV (fL)	46.7 ± 1.3	47.3 ± 0.8
MCH (pg)	14.0 ± 0.8	14.4 ± 0.6
MCHC (g/dL)	30.0 ± 1.3	30.3 ± 1.2
PLT (x10^4/μL)	110.3 ± 16.7	96.5 ± 7.3

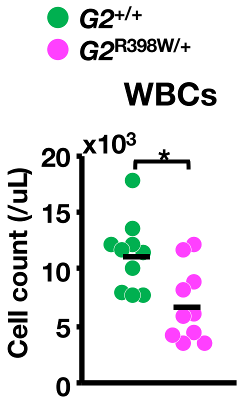

b

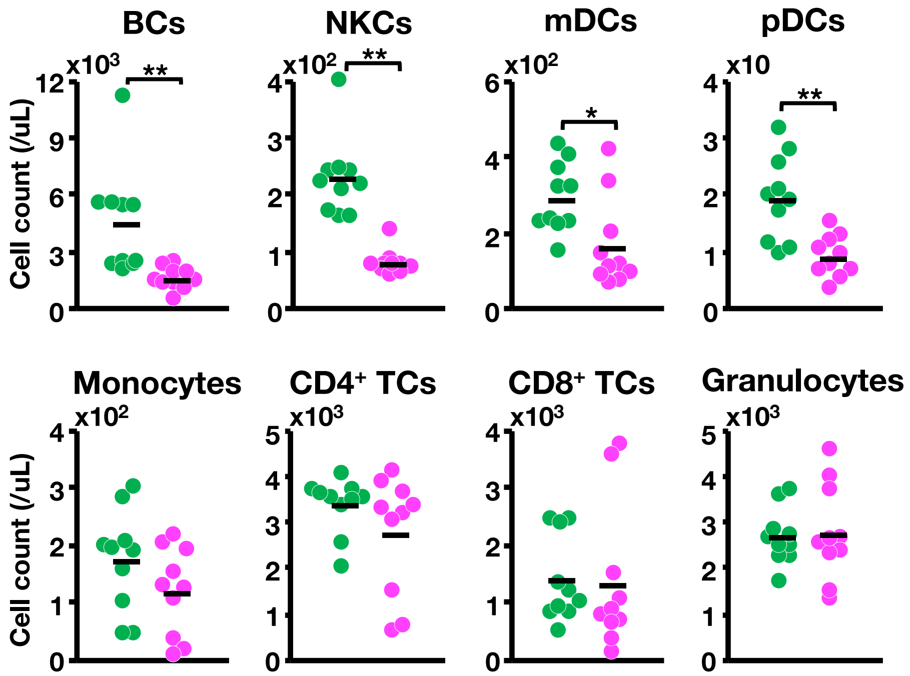

Evaluation of the peripheral blood counts of *G2*$^{R398W/+}$ mice at 5-7 months old.

a Summary of hematopoietic indices. A dot plot of WBC counts in the peripheral blood of *G2*$^{+/+}$ and *G2*$^{R398W/+}$ mice is shown in the right panel. **b** Dot plots of the cell counts of the indicated cell populations of *G2*$^{+/+}$ and *G2*$^{R398W/+}$ mice. *: $p < 0.05$, ** $p < 0.01$. N = 10 of each genotype.

Supplementary Figure 3

a

(N = 6)	*G2*$^{+/+}$	*G2*$^{R398W/+}$
WBC (x10^2/μL)	99.0 ± 14.6	102.0 ± 20.3
RBC (x10^4/μL)	954.3 ± 41.5	954.0 ± 62.5
HGB (g/dL)	14.4 ± 0.5	14.5 ± 0.6
HCT (%)	45.9 ± 3.04	46.2 ± 3.5
MCV (fL)	48.1 ± 1.34	48.5 ± 0.9
MCH (pg)	15.1 ± 0.22	15.2 ± 0.46
MCHC (g/dL)	31.4 ± 1.31	31.5 ± 1.4
PLT (x10^4/μL)	81.0 ± 14.3	84.5 ± 14.6

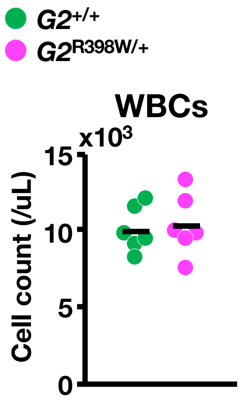

b

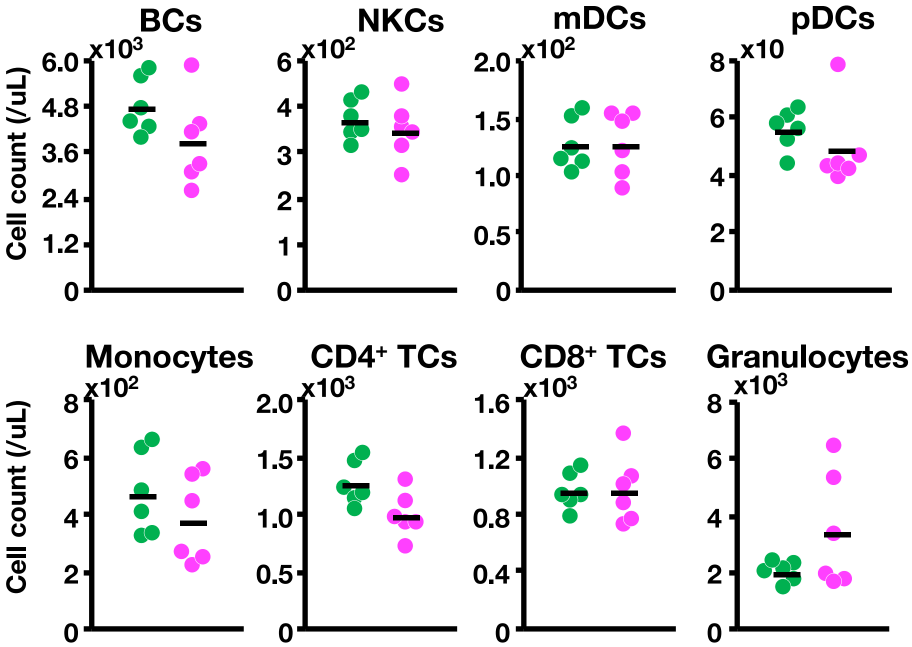

Evaluation of the peripheral blood counts of 3-month-old *G2*$^{R398W/+}$ mice.

a Summary of the hematopoietic indices. A dot plot of the WBC counts in the peripheral blood of *G2*$^{+/+}$ and *G2*$^{R398W/+}$ mice is shown in the right panel. **b** Dot plots of the cell counts of the indicated cell populations of *G2*$^{+/+}$ and *G2*$^{R398W/+}$ mice. N = 6 of each genotype.

Supplementary Figure 4

a

(N=4)	*G2*$^{+/+}$	*G2*$^{R398W/+}$
WBC ($x10^2/\mu L$)	114.8 ± 25.5	79.8 ± 22.8
RBC ($x10^4/\mu L$)	947.3 ± 43.6	983.3 ± 20.4
HGB (g/dL)	13.2 ± 0.8	14.2 ± 0.3
HCT (%)	44.6 ± 3.1	46.5 ± 0.3
MCV (fL)	47.0 ± 2.2	47.3 ± 1.0
MCH (pg)	13.9 ± 0.6	14.5 ± 0.2
MCHC (g/dL)	29.6 ± 1.5	30.6 ± 0.5
PLT ($x10^4/\mu L$)	98.1 ± 12.5	105.9 ± 8.2

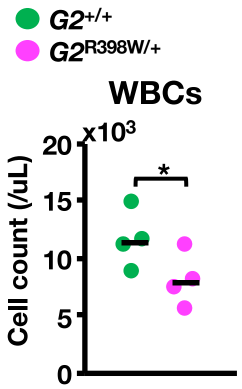

b

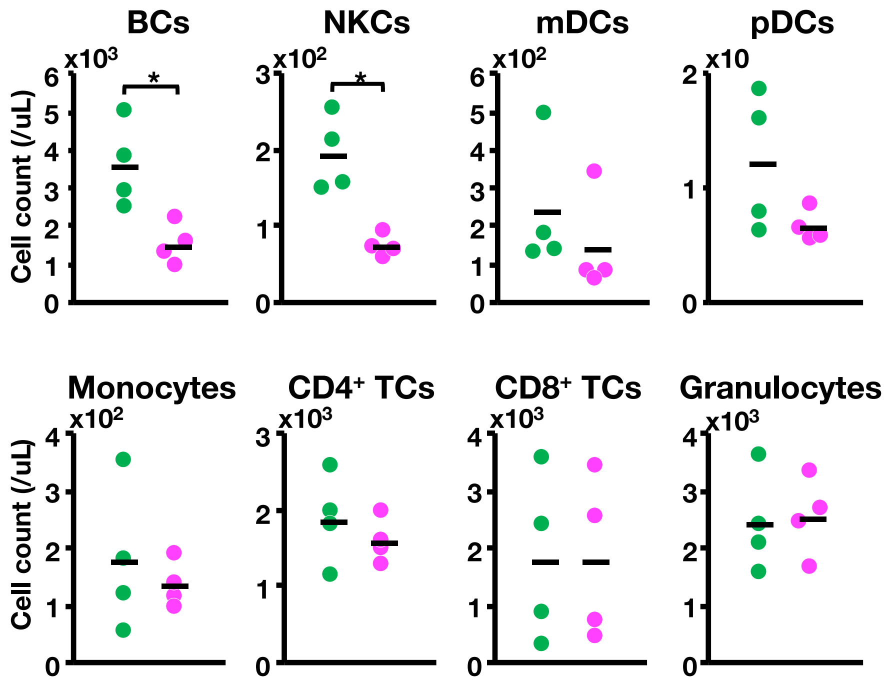

Evaluation of the peripheral blood counts of 6-month-old *G2*$^{R398W/+}$ mice in the 2nd line.

a Summary of hematopoietic indices. A dot plot of WBC counts in the peripheral blood of *G2*$^{+/+}$ and *G2*$^{R398W/+}$ mice is shown in the right panel. **b** Dot plots of the cell counts of the indicated cell populations of the *G2*$^{+/+}$ and *G2*$^{R398W/+}$ mice. *: $p < 0.05$; N = 4 of each genotype.

Supplementary Figure 5

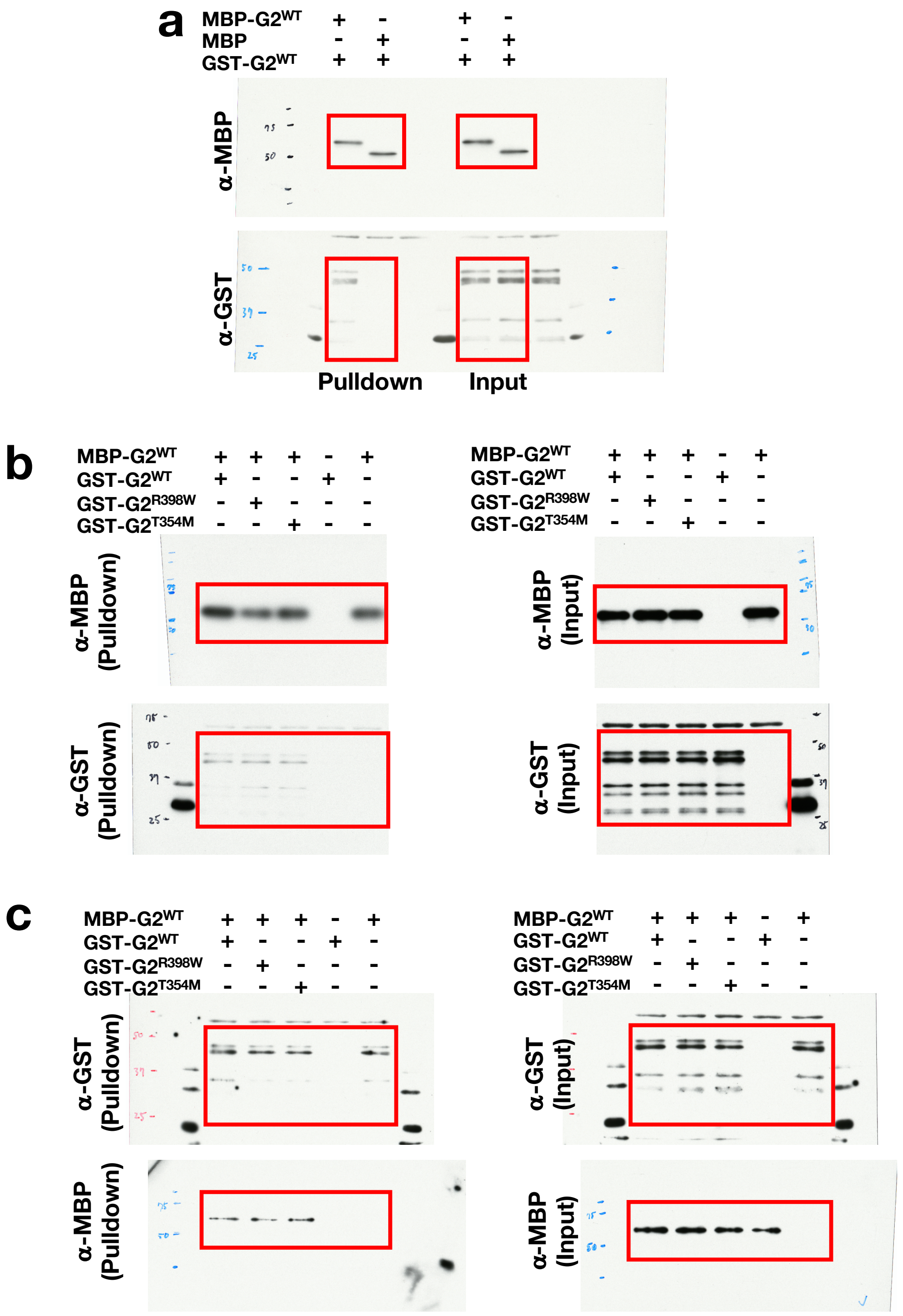

Uncut blots for Figure 6b, 6c and 6d.

a Uncut blots for pull-down assay of MBP-fused G2^{WT}. Images used for Figure 6b are shown in red squares. **b** Uncut blots for pull-down assay of MBP-fused G2^{WT}. Images used for Figure 6c are shown in red squares. **c** Uncut blots for pull-down assay of GST-fused G2^{WT}. Images used for Figure 6d are shown in red squares.

Supplementary Figure 6

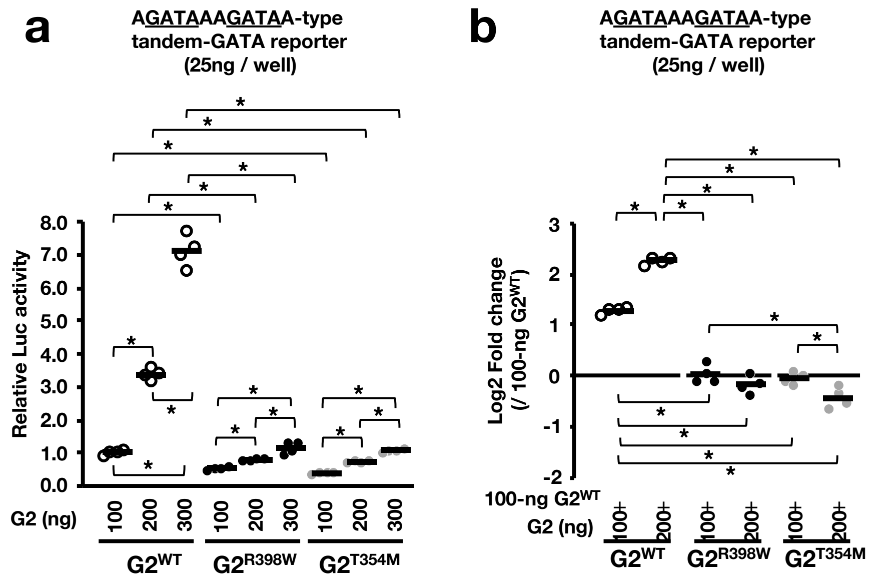

Luciferase reporter analysis using 25 ng of AGATAAGATAA-type tandem-GATA reporter. Scatter dot plots of the transcriptional activity measured by luciferase reporter assay in HEK293T cells using 25 ng of AGATAAGATAA-type tandem-GATA reporter construct together with the respective GATA2 expression constructs at doses of 100, 200 and 300 ng/well (**a**) and together with the 100 ng of the G2^{WT} expression construct and G2^{WT}, G2^{R398W} or G2^{T354M} expression constructs at doses of 0, 100 and 200 ng/well (**b**). The average luciferase activity of G2^{WT} at a dose of 100 ng/well was set to 1.0 in (**a**), and fold changes of the average value of 100 ng of G2^{WT} are shown in (**b**). n=4 biologically independent samples. *: $p < 0.05$.

Supplementary Figure 7

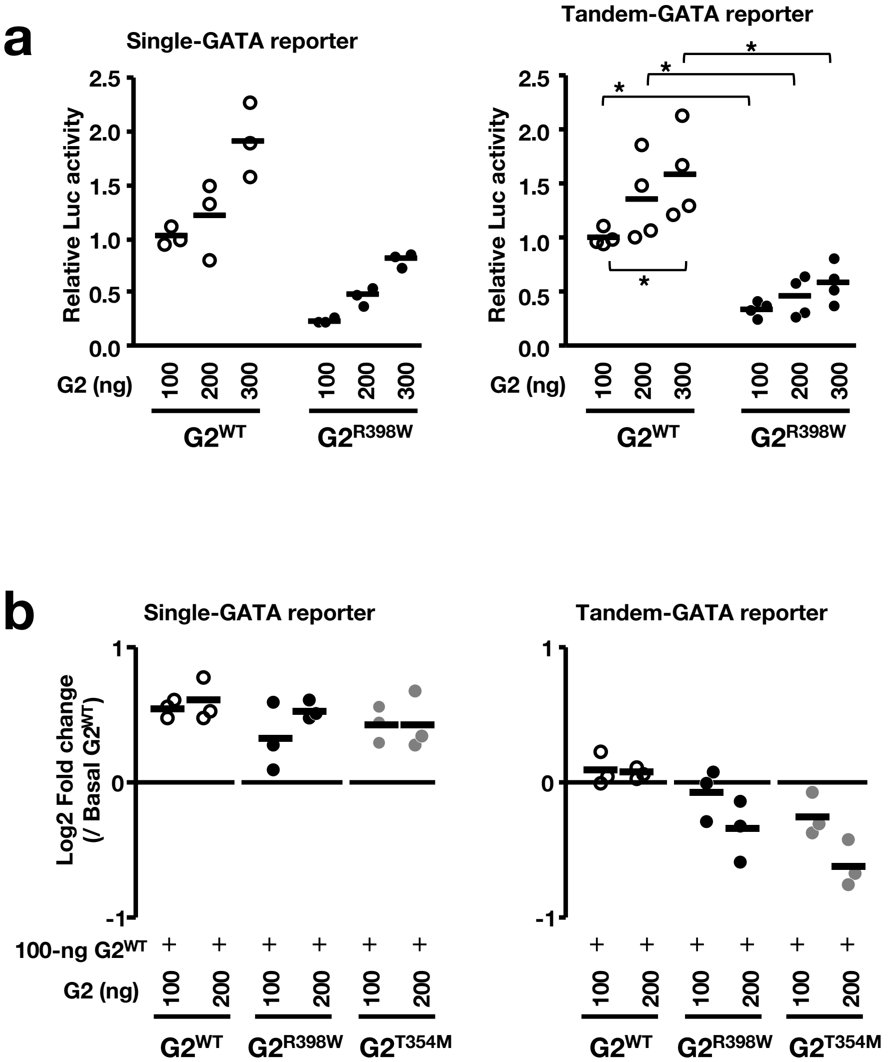

Luciferase reporter analysis using HEK293T cells stably carrying a reporter construct.

a Scatter dot plots of transcriptional activity (n = 3 or 4 per group) measured by luciferase reporter assay in HEK293T cells stably carrying luciferase reporter genes containing single (left) and tandem (right) GATA motifs following transfection with the respective GATA2 expression constructs at doses of 100, 200 and 300 ng/well. The average luciferase activity of $G2^{WT}$ at a dose of 100 ng/well was set to 1.0. **b** A similar set of experiments in which 100 ng of the $G2^{WT}$ expression construct was concomitantly transfected with the $G2^{WT}$, $G2^{R398W}$ or $G2^{T354M}$ expression construct at doses of 0, 100 and 200 ng/well. The average luciferase activity of $G2^{WT}$ at a dose of 100 ng/well was set to 1.0 in (**a**), and fold changes compared to the average value of 100 ng of $G2^{WT}$ are shown in (**b**). * $p < 0.05$.

Supplementary Table 1

List of genes identified in the GATA2 ChIP-seq datasets, which harbor tandem GATA motifs within ±100 kbp of the TSS.

Gene name	Source	Gene name	Source	Gene name	Source
Abcg2	E	*Dnajc5*	A	*Ocln*	A
Acan	A	*Dyrk2*	A	*Olfr1380*	A
Acsl4	E	*Eef2k*	E	*Parvb*	A
Acvrl1	A	*F2rl1*	A	*Polg*	E
Adamts20	A	*Fam210a*	A	*Ppp1r10*	E
Adgrl2	A	*Fam65a*	E	*Prex1*	A
Adipoq	A	*Fam69a*	E	*Pstpip2*	A
Ago2	A, E	*Far2*	A	*Ptpn1*	A
Ahi1	E	*Fcer2a*	A	*Pxmp2*	A
Aldh9a1	A	*Flt1*	A	*Rasgrp4*	A
Amigo2	A	*Gfi1*	A, E	*Rbbp6*	A
Ap5s1	E	*Gnl2*	A	*Rbms1*	A, E
Apol6	A	*Gpr161*	E	*Rem2*	A
Arhgap25	E	*Gramd2*	A	*Rgs10*	E
Arsb	A	*Gsr*	A	*Rpe*	A
Arv1	A, E	*Gtpbp1*	A	*Rsu1*	E
Asph	A	*Hmgb3*	A	*Runx1*	A
Atp8b1	A	*Hs6st1*	A, E	*Selenbp1*	E
B3gnt2	E	*Idh2*	A	*Sema4b*	E
B9d2	A	*Il1rl1*	E	*Skil*	A
Bahd1	A	*Insr*	A	*Slc15a1*	A
Bcor	A	*Irf2*	A	*Smad5*	A, E
Cab39	E	*Itk*	A	*Snx18*	A
Camk1d	A	*Jdp2*	A	*Snx9*	A
Casd1	A	*Jun*	A	*Spin1*	A
Cbfa2t3	A	*Kif3a*	A	*Spry1*	A
Ccr5	A	*Lama1*	A	*Srgap3*	A
Cd24a	A	*Limk2*	A	*Sult6b2*	A
Cd34	A	*Lonp2*	E	*Sync*	E
Cd82	A	*Lypd6*	A	*Taldo1*	A
Chd9	A	*Map3k1*	A, E	*Tbxas1*	E
Chrdl1	A	*Map4k1*	A, E	*Tcte2*	A
Cipc	A	*Mbnl1*	A	*Tle4*	A
Ckap4	E	*Mbp*	A	*Tmem64*	E
Clasp1	A	*Mdga1*	A, E	*Tnfaip3*	A
Clca1	A	*Mrto4*	A	*Traf4*	E
Clk1	A	*Mrvil1*	E	*Traf6*	A
Cpox	A, E	*Mtap*	A	*Ttc32*	A
Crhbp	E	*Myh3*	A	*Ugcg*	A
Cxcr4	A	*Myo5c*	A	*Vcl*	A
Dapk1	A	*Ncs1*	A	*Vmn2r95*	A
Dbx2	E	*Nmu*	A	*Zbp414*	E
Depdc1b	A	*Nxpe5*	A	*Zfp974*	A
Dnahc2	E	*Nyx*	A	*mt-Cytb*	A

A; ChIP Atlas
E; ENCODE

Supplementary Table 2

Primer sequences for genotyping PCR.

	Forward	Reverse
G2^{R398W} allele	TCACCCTGGGGATACAATTAAC	GATTTGCTGGACATCTTCCA
G2-Gfp knockin-knockout allele	CTGAAGTTCATCTGCACCACC	GAAGTTGTACTCCAGCTTGTGC

Supplementary Table 3

Combinations of fluorescently labeled antibodies for flow cytometry.

	Target population	Antigen	Fluorescence label	Manufacturer
Peripheral blood	B cell and T cell	CD4	Fluorescein isothiocyanate (FITC)	BD Pharmingen
		CD8	Phycoerythrin (PE)	BioLegend
		B220	PE-Cyanin 7 (PE-Cy7)	eBioscience
		CD19	Allophycocyanin (APC)	eBioscience
	NK cell	CD49b	Alexa Fluor 488	BioLegend
		CD1d	PE	BioLegend
		CD11b	PE-Cy7	eBioscience
		NKG2D	APC	BioLegend
		CD3	APC-Cy7	BD Biosciences
		NK1.1	Brilliant Violet 421 (BV421)	BD Biosciences
	Myeloid and dendritic cell	CD11b	FITC	eBioscience
		F4/80	APC	eBioscience
		Gr1	APC-eFluor 780 (APC-eF780)	eBioscience
		CD11c	Pacific Blue	BioLegend
Bone marrow	Hematopoietic stem/progenitor cell	CD48	FITC	BioLegend
		CD150	PE	BioLegend
		CD150	BV510	BioLegend
		Flk2	PE	BioLegend
		CD16/32	PE-Cy7	BioLegend
		CD34	Alexa Fluor 647	BioLegend
		cKit	APC-eF780	eBioscience
		Sca1	BV421	BioLegend

Supplementary Table 4

Combinations of fluorescently labeled antibodies for flow cytometry of cells from *Gfp* knockin mice.

	Target population	Antigen	Fluorescence label	Manufacturer
Peripheral blood	B cell and T cell	CD4	APC-eF780	eBioscience
		CD8	PE	BioLegend
		B220	PE-Cy7	eBioscience
		CD19	APC	eBioscience
	NK cell	CD1d	PE	BioLegend
		CD11b	PE-Cy7	eBioscience
		NKG2D	APC	BioLegend
		CD3	APC-Cy7	BD Biosciences
		NK1.1	BV421	BD Biosciences
	Myeloid and dendritic cell	CD11b	PE	eBioscience
		F4/80	APC	eBioscience
		Gr1	APC-eF780	eBioscience
		CD11c	Pacific Blue	BioLegend

Supplementary Table 5

Primer sequences for quantitative RT-PCR.

	Forward	Reverse	Information source
Gapdh	GTCGTGGAGTCTACTGGTGTCTT	GAGATGATGACCCTTTTGGC	
Total *Gata2*	ACCTGTTGTGCAAATTGTCAG	TCTGGATCCCTTCCTTCTTC	
$G2^{R398W}$	GCAAATTGTCAGACGACAACC	GATTTGCTGGACATCTTCCA	
Acvrl1	GGGCCTTTTGATGCTGTCG	TGGCAGAATGGTCTCTTGCAG	PrimerBank
Ago2	GCCGTCCTTCCCACTACCAC	GGTATTGACACAGAGCGTGTGC	ref. 1
Bcor	CTTTCTGCAACCCCTCTGTATG	ACTTGTTCCACACATGCGGA	PrimerBank
Eef2k	CGCTTTGTACCGGGGATTCT	AAGGATGGTCCTCCCACAGT	ref. 2
Gfi1	AGAAGGCGCACAGCTATCAC	GGCTCCATTTTCGACTCGC	ref. 3
Gtpbp1a	GCTGGTTCTAGTGAGCCCTAC	CAGTCCCATCTGATCCCTGC	PrimerBank
Il6ra	CCTGAGACTCAAGCAGAAATGG	AGAAGGAAGGTCGGCTTCAGT	PrimerBank
Jdp2	AGCTGAAATACGCTGACATCC	CTCACTCTTCACGGGTTGGG	PrimerBank
Limk2	GGGCTGTGGCACCTATGTTC	CCAGTTGGTGAGGGATTCCTG	PrimerBank
Map4k1	ATTGGGACACCGTACTGGAT	TGTCGCACAGCTCATTGTAT	ref. 4
Rasgrp4	GGGAAATCAGCAAGGTCATGG	CGGATACATTCCTCCAATAGCTC	PrimerBank
Smad5	TTGTTCAGAGTAGGAACTGCAAC	GAAGCTGAGCAAACTCCTGAT	ref. 5
Stat3	CAATACCATTGACCTGCCGAT	GAGCGACTCAAACTGCCCT	PrimerBank
Tal1	CACTAGGCAGTGGGTTCTTTG	GGTGTGAGGACCATCAGAAATCT	PrimerBank
Tmem56	CAGTCTGTCTGGATCGTTACTTG	AGTTTCCCGTTCTGAAGGCTAT	PrimerBank
Zfpm1	ATCCCCTGAGAGAGAAGAACCG	GGCGTCATCCTTCCTGTAGATC	ref. 6

Supplementary References

1. García-López, J. & del Mazo, J. Expression dynamics of microRNA biogenesis during preimplantation mouse development. *Biochim. Biophys. Acta.* 1819, 847-854 (2012).

2. Jan, A. et al. Activity of translation regulator eukaryotic elongation factor-2 kinase is increased in Parkinson disease brain and its inhibition reduces alpha synuclein toxicity. *Acta. Neuropathol. Commun.* 6, 54 (2018).

3. Nakanishi, Y. et al. Control of paneth cell fate, intestinal inflammation, and tumorigenesis by PKCλ/ι. *Cell Rep.* 16, 3297-3310 (2016).

4. Lee, J. S., Lee, H. J., Lee, J. W., Lee, S. C. & Heo, J. S. Osteogenic effect of inducible nitric oxide synthase (iNOS)-loaded mineralized nanoparticles on embryonic stem cells. *Cell Physiol. Biochem.* 51, 746-762 (2018).

5. Lou, C. H. et al. Posttranscriptional control of the stem cell and neurogenic programs by the nonsense-mediated RNA decay pathway. *Cell Rep.* 6, 748-764 (2014).

6. Welch, J. J. et al. Global regulation of erythroid gene expression by transcription factor GATA-1. *Blood*. 104, 3135-3147 (2004).
