# Supplementary material for: Heterozygous variants in GATA2 contribute to DCML deficiency in mice by disrupting tandem protein binding
Source: Commun Biol. 2022 Apr 19;5:376. doi: 10.1038/s42003-022-03316-w (PMC9018821; doi:10.1038/s42003-022-03316-w)
Supplement: Supplementary file 2 — Description of Additional Supplementary Files [file 42003_2022_3316_MOESM2_ESM.pdf]

Description of Additional Supplementary Files

File name: Supplementary Data 1
Description: Original source data for the Fig 1c, 1g, and 6b-d.
