# Supplementary material for: Heterozygous variants in GATA2 contribute to DCML deficiency in mice by disrupting tandem protein binding
Source: Commun Biol. 2022 Apr 19;5:376. doi: 10.1038/s42003-022-03316-w (PMC9018821; doi:10.1038/s42003-022-03316-w)
Supplement: Supplementary file 4 — Reporting Summary [file 42003_2022_3316_MOESM4_ESM.pdf]

nature portfolio

Corresponding author(s): Ritsuko Shimizu
Last updated by author(s): Mar 20, 2022

Reporting Summary

Nature Portfolio wishes to improve the reproducibility of the work that we publish. This form provides structure for consistency and transparency in reporting. For further information on Nature Portfolio policies, see our Editorial Policies and the Editorial Policy Checklist.

Statistics

For all statistical analyses, confirm that the following items are present in the figure legend, table legend, main text, or Methods section.

n/a	Confirmed	
☐	☒	The exact sample size (*n*) for each experimental group/condition, given as a discrete number and unit of measurement
☐	☒	A statement on whether measurements were taken from distinct samples or whether the same sample was measured repeatedly
☐	☒	The statistical test(s) used AND whether they are one- or two-sided<br>*Only common tests should be described solely by name; describe more complex techniques in the Methods section.*
☒	☐	A description of all covariates tested
☒	☐	A description of any assumptions or corrections, such as tests of normality and adjustment for multiple comparisons
☒	☐	A full description of the statistical parameters including central tendency (e.g. means) or other basic estimates (e.g. regression coefficient) AND variation (e.g. standard deviation) or associated estimates of uncertainty (e.g. confidence intervals)
☐	☒	For null hypothesis testing, the test statistic (e.g. *F*, *t*, *r*) with confidence intervals, effect sizes, degrees of freedom and *P* value noted<br>*Give P values as exact values whenever suitable.*
☒	☐	For Bayesian analysis, information on the choice of priors and Markov chain Monte Carlo settings
☒	☐	For hierarchical and complex designs, identification of the appropriate level for tests and full reporting of outcomes
☒	☐	Estimates of effect sizes (e.g. Cohen's *d*, Pearson's *r*), indicating how they were calculated

Our web collection on statistics for biologists contains articles on many of the points above.

Software and code

Policy information about availability of computer code

Data collection	The protocols have been described in the "Methods" section, including BD FACSAria II, BD FACSDiva software (Becton Dickinson),Biacore-X100 instrument (GE Healthcare), StepOnePlus Real-Time PCR System (Applied Biosystems), Dual-Luciferase reporter assay system (Promega) with Lumat LB 9507 (Berthold Technologies). Standard, open-source tools used in data collection are described in the "Methods" section.
Data analysis	All computational tools used in data analysis are described in the "Methods" section and are open-sourse tools that have been previously published, including the MEME-ChIP and FIMO programs in MEME Suite and the Genomic Regions Enrichment of Annotations Tool (GREAT). Statistical analyses were performed using JMP Pro 14 and 15 (SAS Institute Inc.).

For manuscripts utilizing custom algorithms or software that are central to the research but not yet described in published literature, software must be made available to editors and reviewers. We strongly encourage code deposition in a community repository (e.g. GitHub). See the Nature Portfolio guidelines for submitting code & software for further information.

Data

Policy information about availability of data

All manuscripts must include a data availability statement. This statement should provide the following information, where applicable:

- Accession codes, unique identifiers, or web links for publicly available datasets
- A description of any restrictions on data availability
- For clinical datasets or third party data, please ensure that the statement adheres to our policy

All data reported in this manuscript will be freely available.
The datasets generated during and/or analyzed during the current study are available form the corresponding author on reasonable request.

Field-specific reporting

Please select the one below that is the best fit for your research. If you are not sure, read the appropriate sections before making your selection.

☒ Life sciences ☐ Behavioural & social sciences ☐ Ecological, evolutionary & environmental sciences

For a reference copy of the document with all sections, see nature.com/documents/nr-reporting-summary-flat.pdf

Life sciences study design

All studies must disclose on these points even when the disclosure is negative.

Sample size	Sample-size was calculated based on the similar study in the field and stated in the figure legends or the methods section.
Data exclusions	No data was excluded for analysis.
Replication	The replication numbers were indicated in the figure legends.
Randomization	Animals were randomly assigned for the different treatment in this study.
Blinding	The investigators were not blinded to group allocation during data collection and analysis.

Reporting for specific materials, systems and methods

We require information from authors about some types of materials, experimental systems and methods used in many studies. Here, indicate whether each material, system or method listed is relevant to your study. If you are not sure if a list item applies to your research, read the appropriate section before selecting a response.

Materials & experimental systems

n/a	Involved in the study	
☐	☒	Antibodies
☐	☒	Eukaryotic cell lines
☒	☐	Palaeontology and archaeology
☐	☒	Animals and other organisms
☒	☐	Human research participants
☒	☐	Clinical data
☒	☐	Dual use research of concern

Methods

n/a	Involved in the study	
☒	☐	ChIP-seq
☐	☒	Flow cytometry
☒	☐	MRI-based neuroimaging

Antibodies

Antibodies used	The detailed information of these antibodies has been introduced in this study and supplementary table 3 and 4.
Validation	All of the used antibodies are from the indicated commercial sources in the supplementary table 3 and 4.

Eukaryotic cell lines

Policy information about cell lines

Cell line source(s)	HEK293T cells were used for luciferase reporter assays.
Authentication	No authentication was performed on this cell line.
Mycoplasma contamination	This cell line was tested to be mycoplasma-free.
Commonly misidentified lines (See ICLAC register)	This cell line was not listed as commonly misidentified lines registered on ICLAC.

Animals and other organisms

Policy information about studies involving animals; ARRIVE guidelines recommended for reporting animal research

Laboratory animals	The detailed information of mice has been introduced in this study.

Wild animals	No wild animal was used in this study.
Field-collected samples	The study did not involve samples collected from the field.
Ethics oversight	Animal care and procedures was conducted in accordance with institutional guidelines approved by Animal Experiment Committee of Tohoku University. Experiments were carried out in accordance with the Regulation for Animal Experiments in Tohoku University.

Note that full information on the approval of the study protocol must also be provided in the manuscript.

Flow Cytometry

Plots

Confirm that:

☒ The axis labels state the marker and fluorochrome used (e.g. CD4-FITC).

☒ The axis scales are clearly visible. Include numbers along axes only for bottom left plot of group (a 'group' is an analysis of identical markers).

☐ All plots are contour plots with outliers or pseudocolor plots.

☒ A numerical value for number of cells or percentage (with statistics) is provided.

Methodology

Sample preparation	The detailed information of flow cytometry analyses have been introduced in this study
Instrument	BD FACSAria II
Software	BD FACSDiva software
Cell population abundance	Details are in the methods section
Gating strategy	See methods for details and the figure.

☒ Tick this box to confirm that a figure exemplifying the gating strategy is provided in the Supplementary Information.
